# Horizontal Transfer and Recombination Fuel Ty4 Retrotransposon Evolution in *Saccharomyces*

**DOI:** 10.1093/gbe/evaf004

**Published:** 2025-01-09

**Authors:** Jingxuan Chen, David J Garfinkel, Casey M Bergman

**Affiliations:** Institute of Bioinformatics, University of Georgia, 120 E. Green St., Athens, GA, USA; Department of Biochemistry and Molecular Biology, University of Georgia, 120 E. Green St., Athens, GA, USA; Institute of Bioinformatics, University of Georgia, 120 E. Green St., Athens, GA, USA; Department of Genetics, University of Georgia, 120 E. Green St., Athens, GA, USA

**Keywords:** genome evolution, horizontal transfer, recombination, retrotransposon, yeast

## Abstract

Horizontal transposon transfer (HTT) plays an important role in the evolution of eukaryotic genomes; however, the detailed evolutionary history and impact of most HTT events remain to be elucidated. To better understand the process of HTT in closely related microbial eukaryotes, we studied Ty4 retrotransposon subfamily content and sequence evolution across the genus *Saccharomyces* using short- and long-read whole genome sequence data, including new PacBio genome assemblies for two *Saccharomyces mikatae* strains. We find evidence for multiple independent HTT events introducing the Tsu4 subfamily into specific lineages of *Saccharomyces paradoxus*, *Saccharomyces cerevisiae*, *Saccharomyces eubayanus*, *Saccharomyces kudriavzevii* and the ancestor of the *S. mikatae*/*Saccharomyces jurei* species pair. In both *S. mikatae* and *S. kudriavzevii*, we identified novel Ty4 clades that were independently generated through recombination between resident and horizontally transferred subfamilies. Our results reveal that recurrent HTT and lineage-specific extinction events lead to a complex pattern of Ty4 subfamily content across the genus *Saccharomyces*. Moreover, our results demonstrate how HTT can lead to coexistence of related retrotransposon subfamilies in the same genome that can fuel evolution of new retrotransposon clades via recombination.

**Significance statement**

Transposable elements typically replicate selfishly within the genomes of most species to increase their copy number; however, the frequency and consequences of transposable element movement across species boundaries is less clear. Here, we investigate horizontal transfer of the Ty4 family in *Saccharomyces* yeasts and provide evidence for multiple horizontal transfer events involving the Tsu4 subfamily including parallel events into different populations of the same recipient species. Our work demonstrates that when conditions are favorable, horizontal transfer of transposable elements can occur more frequently than parsimony would imply, and that recombination between resident and horizontally transferred subfamilies can generate novel transposable element lineages.

## Introduction

Transposable elements (TEs) are mobile, repetitive DNA sequences found in nearly all eukaryotes that impact host genome structure, stability, and function. In multicellular organisms, TEs typically mobilize and replicate within the germ cells of their hosts, and thus are vertically inherited from parents to offspring within the same species (Wells and Feschotte 2020). In contrast, TEs can be transmitted between different species through a process known as horizontal transposon transfer (HTT), which can be mediated by interspecific hybridization or parasitism (Gilbert and Feschotte 2018). HTT offers opportunities to investigate how TEs can evade host control mechanisms, how TEs proliferate in previously naive genomic backgrounds, and how invading TEs interact with resident TE families. With the widespread availability of whole genome sequencing data, HTT has been detected across a growing number of species (Schaack et al. 2010; Wallau et al. 2012; Gilbert and Feschotte 2018; Wallau et al. 2018). Nevertheless, many open questions remain about the consequences that HTT has on the evolution of TEs and their host genomes.

HTT events can be identified using different sources of molecular evolutionary evidence including phylogenetic incongruence between host genomes and TE sequences, unexpectedly high sequence similarity between TEs from divergent species, or the patchy distribution of a TE family across a set of closely related lineages (Wallau et al. 2012; Peccoud et al. 2018). Based on these criteria, an increasing number of HTT events have been identified across the tree of life, with many examples in plants and animals (Dotto et al. 2015). While the number of HTT events detected in fungi is still relatively rare (Dobinson et al. 1993; Daboussi et al. 2002; Novikova et al. 2009; Amyotte et al. 2012; Sarilar et al. 2015), a growing number of HTT events have been identified among *Saccharomyces* yeast species (Liti et al. 2005; Carr et al. 2012; Bergman 2018; Czaja et al. 2020; Bleykasten-Grosshans et al. 2021). As in other taxa, previous studies reporting HTT in yeast have typically focused on detecting their existence. However, the timing, geographic location, identity of donor/recipient lineages, mechanisms of transfer, and consequences of these HTT events remain unknown. Furthermore, because many *Saccharomyces* species have overlapping ranges (Boynton and Greig 2014; David et al. 2024), can form interspecific hybrids (Boynton and Greig 2014; Barbosa et al. 2016; Langdon et al. 2019), and often contain introgressed nuclear genes from other species (Almeida et al. 2014; Barbosa et al. 2016; Peter et al. 2018; Albertin et al. 2018), the biological conditions exist to potentially discover many new HTT events in yeast.

The Ty4 long-terminal repeat (LTR) retrotransposon family is a promising model to understand the process and impact of HTT in *Saccharomyces* yeasts. Ty4 elements—like other members of the Ty1/Copia superfamily—are composed of two overlapping ORFs (*gag* and *pol*) flanked by LTRs (Janetzky and Lehle 1992; Stucka et al. 1992). The founding member of the Ty4 family was first identified in *Saccharomyces cerevisiae* (Stucka et al. 1989) and defined the eponymous Ty4 subfamily. After the initial discovery of the Ty4 subfamily, Neuveglise et al. (2002) described a related subfamily called Tsu4 in the distant congener *Saccharomyces uvarum*. More recently, Bergman (2018) reported the unexpected presence of Tsu4 sequences in *Saccharomyces paradoxus*, *Saccharomyces cerevisiae*, and *Saccharomyces mikatae* and proposed these observations could be explained by HTT events from a donor related to *S. uvarum* or its sister species *Saccharomyces eubayanus*. The strongest evidence for HTT involving the Tsu4 subfamily was found in *S. paradoxus* based on its patchy distribution among divergent *S. paradoxus* lineages, a high similarity between *S. paradoxus* Tsu4 elements and those from *S. uvarum* and *S. eubayanus*, and discordance between the phylogeny of Tsu4 elements and the host tree of *Saccharomyces* species. Bergman (2018) also reported evidence for a potential Tsu4 HTT event involving *S. cerevisiae* based on the presence of one Tsu4 full-length element (FLE) in a single strain (called 245) and its high sequence similarity with the Tsu4 sequences introduced by HTT into *S. paradoxus*. Subsequently, O’Donnell et al. (2023) confirmed the presence of Tsu4 sequences in *S. cerevisiae* in a different strain (called CQS), although it is currently unclear whether Tsu4 in strains 245 and CQS arose from the same or different HTT events. Likewise, Bergman (2018) provided limited evidence for a possible third Tsu4 HTT event in *S. mikatae* based on discordance between the phylogeny of Tsu4 elements and the accepted tree for the genus *Saccharomyces* (Borneman and Pretorius 2015; Peris et al. 2023).

Despite these advances, several important aspects of how HTT events affect the evolution of the Ty4 family in *Saccharomyces* yeasts remain unresolved. First, the samples of *S. cerevisiae* and *S. paradoxus* genomes previously studied did not span the global diversity of these species. Thus, the timing, geographic origin, and number of Tsu4 HTT events in these species is not fully understood. Second, previous inferences about the potential donor species for the Tsu4 HTT event into *S. paradoxus* were limited by the lack of high-quality whole genome assemblies (WGAs) for *S. uvarum* and *S. eubayanus*. For example, the inference that Tsu4 elements from *S. eubayanus* are most closely related to those transferred in *S. paradoxus* (Bergman 2018) was made indirectly using genome data from the interspecific hybrid species *S. pastorianus*, which contains subgenomes from *S. eubayanus* and *S. cerevisiae* (Libkind et al. 2011; Baker et al. 2015; Okuno et al. 2016). Third, evidence for the putative Tsu4 HTT in *S. mikatae* was based on a single element from a highly fragmented draft WGA (Cliften et al. 2003), which are known to have incompletely reconstructed TE sequences (Myers et al. 2000). Finally, the single Ty4 family member identified in a draft genome for *S. kudriavzevii* (which is a key outgroup species to *S. cerevisiae*, *S. paradoxus*, and *S. mikatae*) was found to be in an intermediate phylogenetic position to both the Ty4 and Tsu4 subfamilies (Bergman 2018). These results suggest that additional subfamilies in the Ty4 family remain to be discovered, or that recombination occurred between the Ty4 and Tsu4 lineages. Here, we use large-scale short-read resequencing data and high-quality long-read WGAs from multiple species in the genus *Saccharomyces* to address the impact of HTT on the evolution of the Ty4 family. Our results support a complex model for Ty4 family evolution in yeast that is shaped by recurrent HTT events involving the Tsu4 subfamily, lineage-specific extinction events, and creation of new retrotransposon clades through recombination between pre-existing subfamilies that co-occur in the same species because of HTT.

## Results and Discussion

**Fig. 1.**
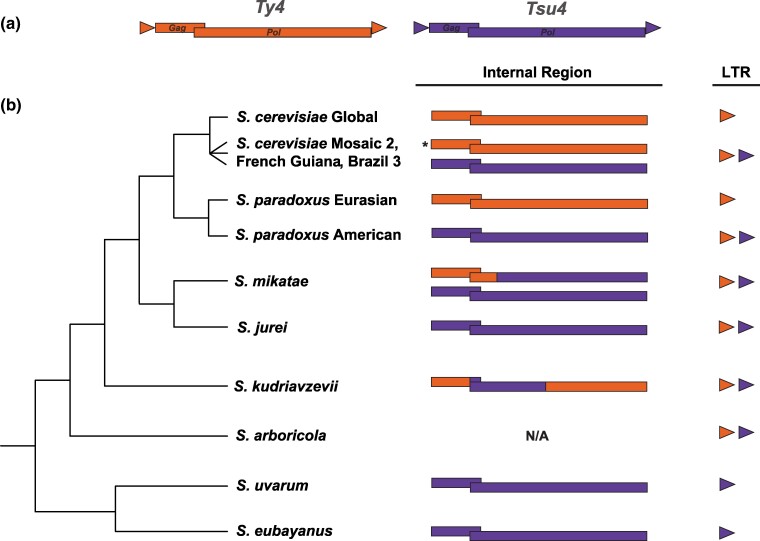
Structure of Ty4 and Tsu4 subfamilies and evolution of subfamily content across the genus *Saccharomyces*. a) Structure of representative pure Ty4 (orange) and Tsu4 (purple) elements defined by *S. cerevisiae* Ty4 and *S. paradoxus* Tsu4 used as query sequences in this study. Both subfamilies have a standard LTR retrotransposon structure comprised of an internal region encoding overlapping Gag and Pol open reading frames flanked by LTRs. b) Schematic phylogeny of *Saccharomyces sensu stricto* species (adapted from Barbosa et al. 2016; Peter et al. 2018; Peris et al. 2023) annotated with LTR and internal sequences of pure and recombinant subfamilies inferred to exist in each species based on analyses in this study. The asterisk for Ty4 internal regions in the Mosaic 2, French Guiana and Brazil 3 *S. cerevisiae* lineages indicates that Ty4 internal sequences have only been found in Mosaic 2 and French Guiana strains.

To better understand the history and impact of HTT events involving the Ty4 family in *Saccharomyces*, we used four complementary genome bioinformatic strategies. First, to more accurately infer the biogeographic distribution and ancestral states of the Ty4/Tsu4 subfamilies in *S. paradoxus* and *S. cerevisiae*, we investigated the presence/absence of Ty4/Tsu4 subfamily sequences across worldwide phylogenies of both species using unassembled short-read WGS datasets. For these analyses, we estimated copy number for LTRs and internal regions separately because recombination between LTRs within FLEs frequently excises internal sequences creating solo LTRs (Farabaugh and Fink 1980). Doing this allows us to interpret the presence of a high copy number of internal sequences as evidence of relatively recent activity, while the presence of LTRs can be interpreted as evidence of either recent or past activity in a strain (Carr et al. 2012). Second, we annotated Ty4/Tsu4 copies in a dataset of over 200 high-quality WGAs for all species in the *Saccharomyces* genus which allowed us to cross-validate Ty4/Tsu4 subfamily presence/absence data based on short-read WGS data and to classify annotated copies at higher resolution into FLEs, truncated elements, and solo LTRs. We interpret the presence of FLEs in a WGA as evidence for recent activity in a strain, while solo LTRs can represent recent and past activity. The relative age of truncated elements is more difficult to interpret since these may arise as artifacts from divergent matches to Ty4/Tsu4 query sequences, or be insertions that are under functional constraint (Hannon-Hatfield et al. 2024). Third, we generated phylogenetic networks and trees for internal coding regions of FLEs extracted from WGAs, which allowed us to directly investigate the molecular evolution of the Ty4 family across the entire *Saccharomyces* genus. Fourth, we generated strain-specific consensus sequences from unassembled short-read WGS datasets, which allowed us to study Tsu4 subfamily evolution among *S. paradoxus*, *S. cerevisiae*, *S. eubayanus*, and *S. uvarum* using larger samples of strains that lack high-quality WGAs. Figure 1 provides an overview of the structures of “pure” elements from the Ty4 and Tsu4 subfamilies used as queries, the phylogeny of the species investigated in this study, and element structures inferred to occur in each species based on the following analyses.

### An Ancestral Tsu4 HTT Event Occurred Prior to Radiation of Indigenous American ***S. paradoxus*** Lineages

**Fig. 2.**
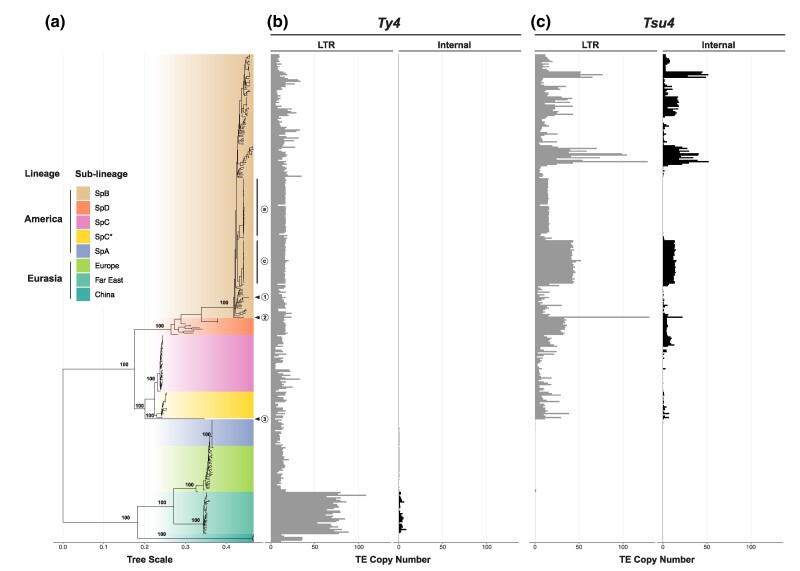
Host phylogeny of *S. paradoxus* annotated with estimated Ty4/Tsu4 copy number. a) Midpoint rooted ML phylogenetic tree of 370 *S. paradoxus* strains integrated from multiple public short-read WGS datasets (see Materials and Methods). Bootstrap support is annotated for key nodes. Major lineages and sub-lineages are annotated according to previously reported population structure (Eberlein et al. 2019; He et al. 2022). The N. American strain DG1768 used in retromobility studies (Moore et al. 2004) is found in the SpB sub-lineage and is indicated by the circled number 1. The S. American strain UFRJ50916 is found in the SpB sub-lineage and is indicated by the circled number 2. The Hawaiian strain UWOPS91-917.1 is found in its own sub-lineage indicated by the circled number 3. Subclades a and c of SpB identified by Xia et al. (2017) are indicated by their respective circled letters. b) Copy number estimates for the Ty4 subfamily. c) Copy number estimates for the Tsu4 subfamily. In b) and c), gray bars represent copy number estimates for LTRs, whereas black bars represent estimated copy number for internal regions.

Using short-read WGS datasets for 370 *S. paradoxus* strains, we reconstructed a maximum likelihood (ML) phylogenetic tree based on 713,556 SNPs that confirmed all major known lineages, sub-lineages, and their relationships (Fig. 2a) (Naumov et al. 1997; Koufopanou et al. 2006; Kuehne et al. 2007; Liti et al. 2009; Leducq et al. 2016; Eberlein et al. 2019; Henault et al. 2019; He et al. 2022). Worldwide diversity in *S. paradoxus* splits into two major lineages: American and Eurasian. The American lineage includes several indigenous North American sub-lineages (SpB, SpC, SpC*, and SpD), as well a lineage with a single strain from Hawaii. SpC* and SpD are hybrid lineages derived from crosses between SpB and SpC, and between SpB and SpC*, respectively (Leducq et al. 2016; Eberlein et al. 2019; Henault et al. 2019). The Hawaiian lineage has been reported to share similarity with either the SpB (Leducq et al. 2014) or SpC/SpC* lineages (He et al. 2022; Peris et al. 2023). Our analysis places the Hawaiian lineage as an outgroup to the SpC/SpC* lineages (circled number 3, Fig. 2a). Importantly, we note that *S. paradoxus* lineage from S. America (circled number 2, Fig. 2a)—which was formerly considered a distinct species called *S. cariocanus* (Naumov et al. 2000)—is contained within the North American SpB sub-lineage (Koufopanou et al. 2006; Liti et al. 2006, 2009; Hyma and Fay 2013; Leducq et al. 2014; He et al. 2022). The Eurasian lineage includes sub-lineages indigenous to Europe, Far East Asia, and China, as well as a sub-lineage (SpA) composed of strains from North America that descend from a recent trans-oceanic migration event (Kuehne et al. 2007; Leducq et al. 2016).

By mapping estimated Ty4/Tsu4 subfamily copy number onto the global phylogeny for *S. paradoxus*, we detected Ty4 LTR sequences in all *S. paradoxus* strains from both the Americas and Eurasia (Fig. 2b). Ty4 LTR copy number is much higher in strains from the Far East sub-lineage (mean±SD: 74.85±9.58) relative to other *S. paradoxus* sub-lineages, which otherwise range from 10.59±2.72 in SpA to 24.21±12.57 in China (Fig. 2b, supplementary fig. S1, Supplementary Material online). Ty4 internal regions are essentially absent across the species, except in the LTR-rich Far East sub-lineage which are estimated to have 2.74±1.88 Ty4 internal regions per strain (Fig. 2b, supplementary fig. S1, Supplementary Material online). In contrast, Tsu4 LTR sequences are only found in indigenous American strains and essentially absent from strains with a Eurasian origin (Fig. 2c, supplementary fig. S1, Supplementary Material online) (see also Henault et al. 2020), including the Eurasian-derived SpA sub-lineage that has remained isolated from indigenous American sub-lineages after secondary contact (Kuehne et al. 2007; Hyma and Fay 2013; Leducq et al. 2014, 2016; He et al. 2022). Among indigenous American lineages, Tsu4 LTR copy number ranges from 9.91±6.51 in SpC to 33.14±2.06 in SpD, with the highest variation among strains within the SpB lineage (24.42±24.05). Tsu4 internal sequences are found in all indigenous American sub-lineages (SpB, SpC, SpC*, SpD, and Hawaii) with variable copy number across strains within sub-lineages (Fig. 2c, supplementary fig. S1, Supplementary Material online). Tsu4 internal copy number ranges from 1.68±1.99 in SpC* to 7.68±10.68 in SpB, with SpB also being the sub-lineage with the highest variation among strains. Estimated Tsu4 copy number across strains varied substantially between two subclades of SpB (called cladeas a and c) previously identified by Xia et al. (2017) that are each comprised of closely related clonal lineages sampled at the same location in Ontario, Canada over consecutive years (Fig. 2c). Subclade a strains have low Tsu4 LTR content (15.2±0.01) and lack Tsu4 internal regions (0±0.01), whereas subclade c strains have high estimated LTR (43.3±2.18) and internal region (13.3±0.88) copy number. These results indicate that Tsu4 copy number can vary substantially among *S. paradoxus* strains at fine spatial scales even within the same sub-lineage, but also that Tsu4 copy number is stable and reproducibly estimated among strains from the same clonal lineage.

To cross-validate results based on short-read WGS data, we analyzed a smaller dataset of 12 WGAs generated using PacBio or Oxford Nanopore Technology (ONT) long-read data that samples all major *S. paradoxus* lineages (supplementary table S1, Supplementary Material online). Ty4 solo LTRs were found in all strains but Ty4 FLEs were only found in the Far East strain N44. In contrast, Tsu4 solo LTRs are identified in the nine indigenous American *S. paradoxus* strains and are absent from the other three strains with Eurasian origin (CBS432, N44, and LL2012_001). At least one Tsu4 FLE is identified in all indigenous American *S. paradoxus* strains with WGAs except for the SpB strain DG1768 that is commonly used in retromobility studies (Chen et al. 2022b) (circled number 1, Fig. 2a). As previously reported (Bergman 2018), Tsu4 FLE copy number in the South American SpB strain UFRJ50916 is much higher (n=22) than other *S. paradoxus* strains with WGAs (n≤4).

These data indicate that the Ty4 subfamily was present in the most recent common ancestor (MRCA) of all *S. paradoxus* lineages prior to global dispersal and therefore represents the ancestral subfamily in *S. paradoxus*. The Ty4 subfamily subsequently went extinct in most recognized *S. paradoxus* sub-lineages except for the Far East sub-lineage where FLEs are present and presumably active. In contrast, the lack of Tsu4 sequences in Eurasian *S. paradoxus* and the Eurasian-derived SpA sub-lineage indicates this subfamily has never existed in Eurasia and therefore was not present in the MRCA of all *S. paradoxus* strains. Our results support the interpretation that a Tsu4 HTT event occurred in an ancestor of all indigenous American *S. paradoxus* sub-lineages after the divergence of American from Eurasian lineages. This HTT event most likely occurred in an ancestral lineage where the Ty4 subfamily had already gone extinct, thus explaining why Ty4 and Tsu4 FLEs have never been observed in the same *S. paradoxus* strain. Since this ancestral HTT event, Tsu4 has maintained activity in all indigenous American *S. paradoxus* sub-lineages. However, Tsu4 has secondarily gone extinct or expanded to very high copy-number in many strains in each American *S. paradoxus* sub-lineage. We note that this parsimonious scenario does not exclude the possibility of additional more recent Tsu4 HTT events into indigenous American *S. paradoxus* lineages that are obscured by this initial ancestral HTT event.

### HTT has Introduced Tsu4 into a Small Number of Central/South American *S. cerevisiae* Strains

**Fig. 3.**
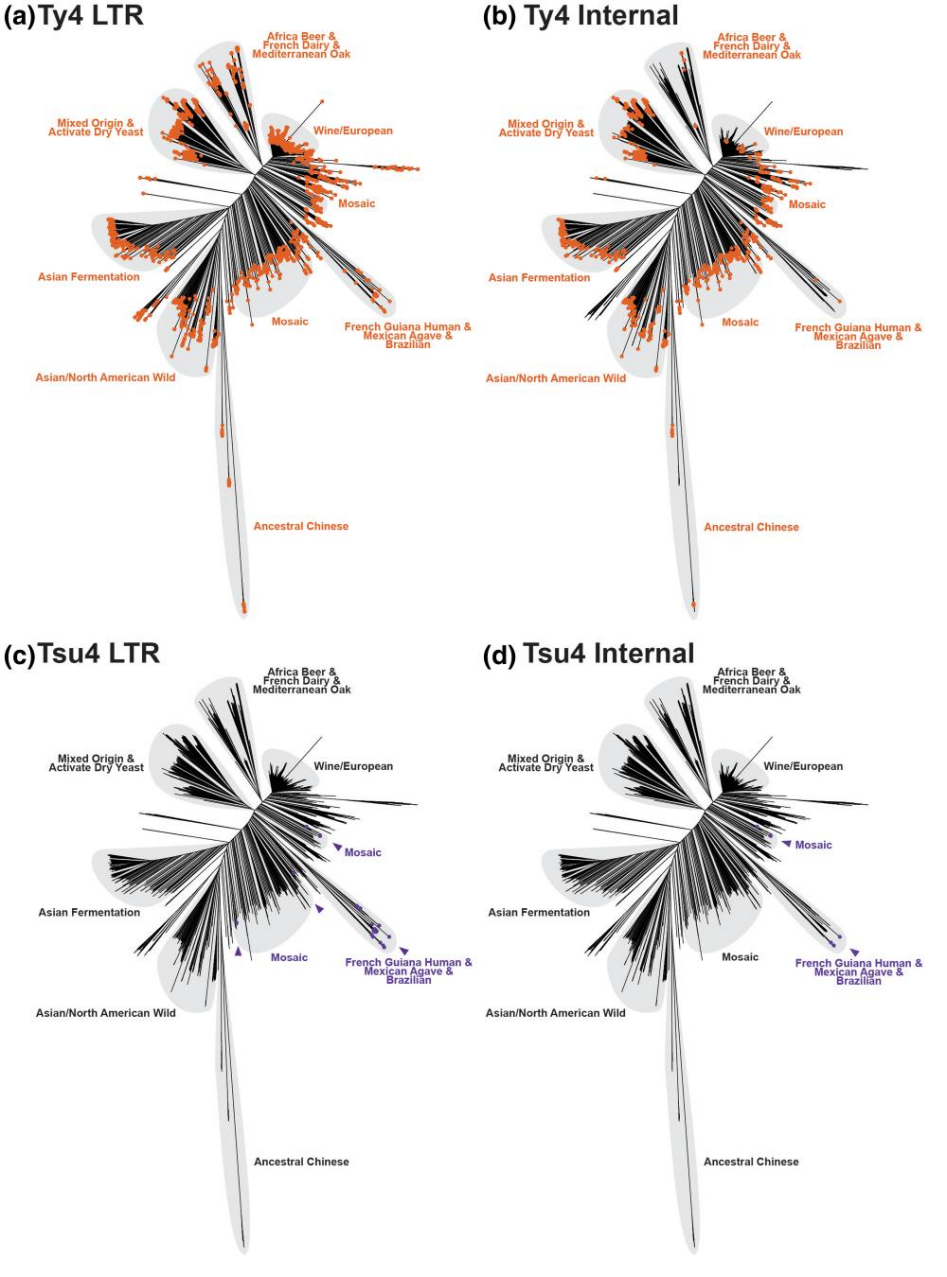
Host phylogeny of *S. cerevisiae* annotated with the presence or absence of Ty4/Tsu4 subfamilies. a–d) NJ phylogenetic tree reconstructed using 2,787,577 genome-wide SNPs from 2,404 *S. cerevisiae* strains (see Materials and Methods). Major lineages are annotated with light gray shading based on previously reported population structure (Duan et al. 2018; Peter et al. 2018). Colored dots indicate the presence of Ty4 (a and b) and Tsu4 (c and d) sequences for each *S. cerevisiae* strain. The presence of Ty4/Tsu4 subfamilies in a strain was inferred when copy number estimates were >1 for LTRs (annotated with orange dots in a and c) and >0.5 for internal regions (annotated with purple dots in b and d).

Using a similar short-read WGS-based approach to that used above for *S. paradoxus*, we reconstructed a species-wide phylogeny for *S. cerevisiae* based on 2,787,577 genome-wide SNPs from 2,404 strains (Fig. 3). In contrast to the ML approach used to reconstruct the phylogeny of *S. paradoxus* where admixture among sub-lineages is rare, we followed Peter et al. (2018) in using a neighbor-joining (NJ) approach to generate the *S. cerevisiae* phylogeny which accommodates the well-established existence of admixed strains in this species (Liti et al. 2009; Peter et al. 2018). Despite using nearly twice as many strains, our phylogenetic tree of *S. cerevisiae* strains shows a similar topology as Peter et al. (2018), who identified a complex population structure including more than 26 distinct lineages plus many mosaic strains derived from admixture between these lineages. Strains in our integrated dataset that are not present in Peter et al. (2018)—such as those from Duan et al. (2018) and Barbosa et al. (2016)—cluster with known lineages previously characterized by Peter et al. (2018). For instance, “activated dry yeast” strains from Duan et al. (2018) cluster in the “mixed origin” lineage from Peter et al. (2018) (Fig. 3).

We then visualized the presence/absence of LTR and internal regions for Ty4/Tsu4 subfamilies on the phylogeny inferred for *S. cerevisiae* from short-read WGS data. This analysis revealed that Ty4 LTR and internal sequences are present in all *S. cerevisiae* lineages (Fig. 3a,b). In contrast, Tsu4 LTR sequences are restricted to ∼2% of strains surveyed (49/2,404) all of which are found in Central/South America (specifically French Guiana, Mexico, Brazil, and the French West Indies) (Fig. 3c,d). Tsu4 sequences are completely absent from most other *S. cerevisiae* lineages, including the most ancestral Chinese lineages (Wang et al. 2012; Duan et al. 2018). We identified six *S. cerevisiae* strains that contained Tsu4 DNA sequence from internal regions (245, AFQ, and CDM from Mosaic lineage 2; CQS from the French Guiana lineage; UFMG-CM-Y641 and UFMG-CM-Y642 from the Brazil 3 lineage) (Marsit et al. 2015; Barbosa et al. 2016; Peter et al. 2018). Three of these *S. cerevisiae* strains (AFQ, CDM, and CQS) also contain internal regions for Ty4.

To confirm results based on short-read WGS data, we analyzed Ty4/Tsu4 subfamily content in WGAs for a global sample of 183 *S. cerevisiae* strains (supplementary table S1 and fig. S2, Supplementary Material online). With the exception of short-read WGAs for the three strains detected to contain Tsu4 internal sequences in WGS data (245, AFQ and CDM), all of these WGAs were based on long-read sequencing data. No WGAs are publicly available for the two Brazil 3 strains (UFMG-CM-Y641 and UFMG-CM-Y642) with evidence of Tsu4 internal sequences based on WGS data. Ty4 subfamily sequences were found in all *S. cerevisiae* WGAs analyzed, while Tsu4 subfamily sequences were absent from the majority of *S. cerevisiae* WGAs. We identified one full-length Tsu4 copy in short-read WGAs for strains 245 and AFQ. CQS is the only strain assembled using long-read data for which we identify FLEs for Tsu4 (n=9), confirming previous observations (O’Donnell et al. 2023). CQS is also the only WGA in which we identify FLEs for both Tsu4 and Ty4 (n=1). All three *S. cerevisiae* strains with Tsu4 FLEs in WGAs (245, AFQ and CQS) are geographically restricted to Central/South America.

The prevalence of the Ty4 subfamily in most *S. cerevisiae* lineages—including ancestral Chinese lineages (Wang et al. 2012; Duan et al. 2018)—indicates that the Ty4 subfamily was present in the MRCA of this species. However, despite being broadly active at the species level, the absence of Ty4 internal regions and FLEs in many strains indicates this subfamily has undergone many local extinction events (see also Bleykasten-Grosshans et al. 2021). In contrast, the absence of Tsu4 in most lineages (including ancestral Chinese lineages) strongly indicates that this subfamily was not present in the MRCA of *S. cerevisiae*. The small number of strains that do contain Tsu4 in *S. cerevisiae* do not form a single monophyletic group, which is consistent either with one HTT event followed by admixture among lineages, or multiple independent HTT events that have introduced Tsu4 into different lineages of *S. cerevisiae* in Central/South America. Finally, the observation of *S. cerevisiae* strains with FLEs for both Tsu4 and Ty4 subfamilies (i.e. CQS) demonstrates that both subfamilies can co-exist in the same *Saccharomyces* strain and that conditions for recombination between active lineages of Tsu4 and Ty4 exist in nature.

### Multiple HTT Events Have Transferred Tsu4 into *S. paradoxus* and *S. cerevisiae*

The strategies used above allowed us to establish Ty4 as the ancestral subfamily in *S. paradoxus* and *S. cerevisiae*, and to identify at least one Tsu4 HTT event in both species. However, these approaches cannot resolve how many Tsu4 HTT events occurred in either species, nor can they identify the potential donor lineages for these HTT events. To investigate whether the presence of Tsu4 in *S. paradoxus* and *S. cerevisiae* can be explained by one or more Tsu4 HTT event, and to identify the most likely donor lineage(s) for these HTT events, we analyzed the molecular evolution of all Ty4 family FLEs identified in a integrated dataset of 210 high-quality WGAs for all recognized species in the genus *Saccharomyces*. The majority of WGAs in this dataset were generated using long reads, with the exception of short-read WGAs for the three *S. cerevisiae* strains with Tsu4 internal sequences mentioned above (245, AFQ, and CDM) and for two strains of *S. arboricola* (H-6 and ZP960) that represent the best available WGAs for this species. This dataset also includes two new high-quality WGAs for *S. mikatae* strains IFO 1815 and NBRC 10994 that we generated using PacBio sequencing (supplementary table S2, Supplementary Material online). Phylogenetic analysis of our new *S. mikatae* WGAs confirmed the taxonomic placement of IFO1815 in the Asia A clade (Peris et al. 2023) and suggests that NBRC 10994 should be placed in a new clade (which we call Asia C) (supplementary fig. S3, Supplementary Material online).

In total, we identified 247 FLEs for the Ty4 subfamily and 124 FLEs for the Tsu4 subfamily in this integrated dataset (supplementary table S2, fig. S2, Supplementary Material online). No FLEs for either subfamily were identified *S. arboricola* and *S. kudriavzevii* using our current query sequences. The absence of Ty4 family FLEs in *S. arboricola* may simply reflect the lack of high-quality WGAs for this species. However, the absence of Ty4 family FLEs in *S. kudriavzevii* is likely an artifact of divergence between our current Tsu4 query sequence from *S. paradoxus* and the active lineage of the Ty4 family in *S. kudriavzevii*. In *S. kudriavzevii* strain (IFO1802), we observed a high copy number of truncated Tsu4 elements (n=8) that had hallmarks of being from a previously unreported active lineage of the Ty4 family. Five of these “truncated” elements were nearly full-length, highly similar to one another, dispersed throughout the IFO1802 genome, and overlapped full-length de novo LTRharvest predictions (Ellinghaus et al. 2008). We concluded that these five *S. kudriavzevii* elements represented FLEs from a novel active lineage in the Ty4 family and included them in our phylogenetic analysis of FLEs.

**Fig. 4.**
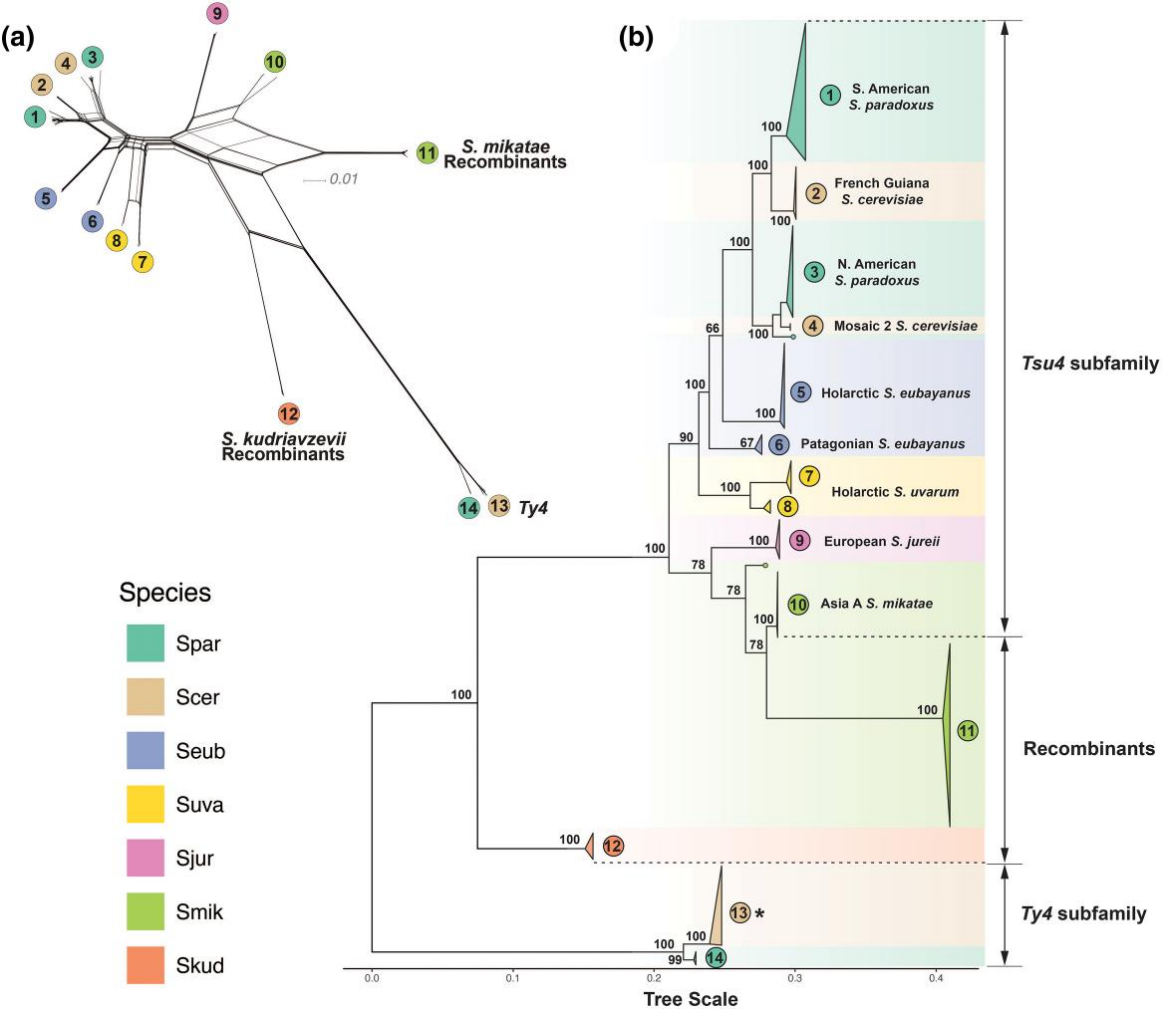
Phylogenetic network and tree of FLEs from the Ty4 family in *Saccharomyces*. a) Phylogenetic network for internal coding regions of Ty4/Tsu4 FLEs based on the NeighborNet algorithm. To simplify visualization, this network only includes *S. cerevisiae* Ty4 subfamily FLEs from WGAs reported in Yue et al. (2017). Lineages in the network are labeled according to monophyletic groups identified in b. b) Midpoint rooted ML phylogeny of internal coding regions from Ty4/Tsu4 FLEs. Bootstrap support based on 100 replicates is shown for major nodes. The scale bar for branch lengths is in units of substitutions per site. All monophyletic groups are collapsed as triangles. Two singleton Tsu4 elements (f267 from Hawaiian *S. paradoxus* strain UWOPS91-917.1 and f256 from *S. mikatae* strain NBRC 10994) are denoted as dots at tips. Triangles, tip dots, and ranges are colored for each species. Vertical heights of triangles are proportional to the number of taxa. Horizontal widths of triangles are equal to the maximum branch length within the clade. Note that the monophyletic clade for the Ty4 subfamily from *S. cerevisiae* (annotated with an asterisk) is re-scaled to 5% of the real sample size both horizontally and vertically, due to the large number of Ty4 sequences (n=244) in *S. cerevisiae* genomes.

To understand the evolutionary history and subfamily diversity of the Ty4 family in *Saccharomyces*, we next created a multiple sequence alignment and reconstructed phylogenetic networks and trees based on internal coding regions of all 376 FLEs in our integrated dataset (Fig. 4a,b). We excluded LTR and untranslated sequences from this analysis, which exhibited poor alignment due to higher divergence in noncoding regions. This analysis identified 14 well-supported clades of species-specific FLEs, plus two branches with singleton FLEs from the Hawaiian *S. paradoxus* strain UWOPS91-917.1 and the Asia C *S. mikatae* strain NBRC 10994, respectively. Two clades (Clades 11 and 12) with FLEs from either *S. mikatae* or *S. kudriavzevii* exhibit evidence of reticulation between the Ty4 and Tsu4 clades (Fig. 4a), which we interpret as being caused by recombination between these subfamilies (see detailed analysis below). Exclusion of these clades eliminated the major signal for reticulation between the Ty4 and Tsu4 subfamilies in the phylogenetic network (supplementary fig. S4A, Supplementary Material online) and increased bootstrap support for clades in *S. jurei* and *S. mikatae*, but did not alter the topological relationships of any clades in the ML tree (supplementary fig. S4B, Supplementary Material online).

The Ty4 subfamily is represented by two clades with FLEs from only *S. cerevisiae* (Clade 13) or *S. paradoxus* (Clade 14), respectively. In contrast, the Tsu4 subfamily is represented by ten species-specific clades (1–10) with FLEs from all species except *S. kudriavzevii* (Fig. 4a, supplementary figs S5, S6, and S7, Supplementary Material online). Two clades each of Tsu4 FLEs from *S. paradoxus* (Clades 1 and 3) and *S. cerevisiae* (Clades 2 and 4) plus the singleton FLE from Hawaiian *S. paradoxus* UWOPS91-917.1 together form a monophyletic group. *Saccharomyces eubayanus* is represented by two Tsu4 FLEs clades, one each from the Holarctic (Clade 5) and Patagonian (Clade 6) lineages, respectively. Tsu4 FLEs from *S. uvarum* form two clades (Clades 7 and 8) that are both found in a single strain from the Holarctic lineage. Tsu4 FLEs from European *S. jurei* (Clade 9) cluster with Tsu4 FLEs from the *S. mikatae* Asia A lineage (Clade 10) and the singleton branch from the *S. mikatae* Asia C lineage. We observe evidence for recombination between elements in Tsu4 Clades 7 and 8 (supplementary fig. S4A, Supplementary Material online), which could arise from non-allelic exchange (gene conversion) among paralogous insertions in the same *S. uvarum* strain (CBS 7001) (supplementary fig. S6, Supplementary Material online) (Roeder and Fink 1982). Similarly, we observe evidence for recombination between elements in Tsu4 Clade 10 and recombinant Clade 11 in *S. mikatae* (Fig. 4a), which is plausible since both clades occur in strains IFO 1815 and NBRC 10994 (supplementary fig. S7, Supplementary Material online).

Previous analysis of Tsu4 HTT events in *S. paradoxus* and *S. cerevisiae* using a smaller dataset of FLEs (Bergman 2018) suggested one primary HTT occurred in the ancestor of all American *S. paradoxus* sub-lineages followed by one secondary HTT from *S. paradoxus* into *S. cerevisiae*. This hypothesis predicts that Tsu4 FLEs from *S. paradoxus* and *S. cerevisiae* should form a single clade, with *S. cerevisiae* FLEs forming a single sub-clade somewhere within the broader diversity of *S. paradoxus* FLEs. Two key features of the Tsu4 FLE phylogeny in our expanded dataset are inconsistent with this hypothesis (Fig. 4). First, we observe two distinct clades of FLEs for both *S. paradoxus* and *S. cerevisiae*, whose closest sampled relatives are each from a different species. Namely, *S. cerevisiae* Clade 2 (from the French Guiana strain CQS) clusters with S. American *S. paradoxus* Clade 1, while *S. cerevisiae* Clade 4 (from Mosaic 2 strains 245 and AFQ) clusters with the N. American *S. paradoxus* Clade 3 and the single FLE from Hawaii. Second, the topology of Tsu4 FLEs in *S. paradoxus* does not strictly follow the host phylogeny as shown by the S. American *S. paradoxus* SpB strain UFRJ50816. Specifically, Tsu4 FLEs from UFRJ50816 form their own divergent Clade 1 instead of grouping as expected with Tsu4 FLEs from other N. American SpB strains (MSH-604 and YPS138) in Clade 3 (supplementary fig. S5, Supplementary Material online).

We interpret the phylogeny of Tsu4 FLEs in *S. paradoxus* and *S. cerevisiae* to be consistent with at least two Tsu4 HTT events into each species, all of which occurred in the Americas. In *S. paradoxus*, we infer one more ancestral Tsu4 HTT event creating Clade 3 plus Hawaiian FLEs that are present in all SpB, SpC, SpC*, SpD, and Hawaiian strains, and one more recent Tsu4 HTT event creating Clade 1 FLEs present in the S. American SpB strain UFRJ50816 (where Clade 3 FLEs had already gone extinct). Likewise, in *S. cerevisiae*, we infer one HTT into the French Guiana lineage creating Clade 2 FLEs, and another HTT into the Mosaic 2 lineage creating Clade 4 FLEs. The nesting of Clade 4 within a cluster containing Clade 3 plus Hawaiian FLE group suggests this HTT event was a direct transfer from *S. paradoxus* to *S. cerevisiae* by interspecific mating and backcrossing to *S. cerevisiae*, which is plausible since *S. cerevisiae* Mosaic 2 strains also contain many ORFs introgressed from *S. paradoxus* (Peter et al. 2018). Additionally, the existence of multiple Tsu4 clades in *S. cerevisiae* supports independent Tsu4 HTT events rather than a single event followed by admixture to explain its presence in different *S. cerevisiae* lineages. Finally, these data suggest that when conditions are conducive for HTT in *Saccharomyces* (e.g. sympatry, interspecific hybridization), the number of HTT events that occur may be more than the most parsimonious interpretation based on presence/absence data would imply.

### Tsu4 in *S. paradoxus*, *S. cerevisiae*, and Holarctic *S. eubayanus* Were Transferred from an Unknown Donor

The collective monophyly of all Tsu4 clades found in *S. paradoxus* and *S. cerevisiae* suggests the Tsu4 HTT events in these species ultimately arose from a similar donor lineage, with distinct clades being formed by HTT events that occurred at different times or in different geographic regions. Using indirect data from the hybrid species *S. pastorianus* that contains subgenomes from *S. eubayanus* and *S. cerevisiae* (Libkind et al. 2011; Baker et al. 2015; Okuno et al. 2016), Bergman (2018) concluded that the Holarctic lineage of *S. eubayanus* contains the most closely related Tsu4 sequences to those in *S. paradoxus* and *S. cerevisiae*. Our current analysis provides direct evidence for this conclusion, with Clade 5 FLEs from the Himalayan strain CDFM21L.1 in the Holarctic *S. eubayanus* lineage clustering most closely with the common ancestor of all Tsu4 FLEs in *S. paradoxus* and *S. cerevisiae* (Fig. 4). This result raises the possibility that Holarctic *S. eubayanus* may represent the most likely donor lineage for the multiple HTT events observed in *S. paradoxus* and *S. cerevisiae*.

However, several pieces of evidence suggest that Tsu4 FLE found in the Holarctic *S. eubayanus* strain CDFM21L.1 may not represent the direct donor lineage for the HTT events in *S. paradoxus* and *S. cerevisiae* (Fig. 4). First, all observed HTT events in *S. paradoxus* and *S. cerevisiae* occur in the Americas, while CDFM21L.1 is a central Asian strain. Second, Tsu4 clades in *S. paradoxus* and *S. cerevisiae* are not nested within the diversity of FLEs from Holarctic *S. eubayanus*, but rather form a sister group separated by substantial divergence. Third, bootstrap support for the clustering of Holarctic *S. eubayanus* FLEs with the ancestor of FLEs from *S. cerevisiae* and *S. paradoxus* is relatively weak (>66%). The alternative clustering of FLEs from Holarctic and Patagonia-B *S. eubayanus* lineages together would suggest that the donor into *S. paradoxus* and *S. cerevisiae* is from a currently unsampled lineage of *S. eubayanus*, or a species closely related to *S. eubayanus*. Fourth, Tsu4 FLEs from Holarctic *S. eubayanus* are not nested with in the diversity of *S. eubayanus* Patagonian FLEs (Fig. 4, supplementary fig. S6, Supplementary Material online), as is expected since Holarctic *S. eubayanus* is known to be a sub-lineage of the Patagonia-B lineage (supplementary fig. S8, Supplementary Material online) (Peris et al. 2016). Discordance between the *S. eubayanus* Tsu4 FLE and host strain phylogenies suggests the possibility of a previously undetected Tsu4 HTT event on the *S. eubayanus* lineage leading to CDFM21L.1, which could lead to the false conclusion that the Holarctic *S. eubayanus* lineage is the most likely donor for HTT events into *S. paradoxus* and *S. cerevisiae*.

To test for an undetected Tsu4 HTT event in the Holarctic *S. eubayanus* lineage, we developed a novel approach to study Tsu4 sequence evolution using strain-specific consensus sequences inferred from short-read WGS data. Importantly, this approach bypasses the limited number of WGAs available in *S. eubayanus* and other potential donor species and allows us to generalize results across larger samples of host strains and lineages. The premise behind this approach is based on the observation that Tsu4 FLEs typically cluster first within the same strain before clustering with FLEs from other strains (Fig. 4). This pattern may arise because of turnover of FLE haplotypes through transposition and excision, or because of gene conversion among paralogous insertions. Regardless, this pattern of concerted evolution suggests that intra-strain consensus sequences should be a reasonable proxy for the common ancestor of elements within a strain, and that strain-specific Tsu4 consensus sequences can be used for evolutionary inference across strains and species.

**Fig. 5.**
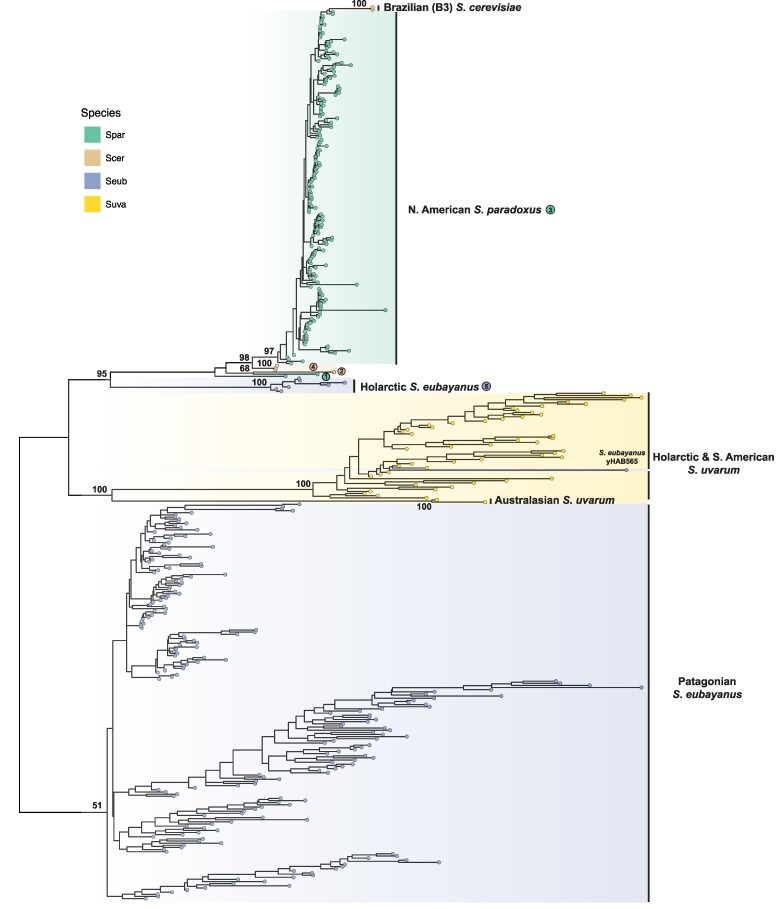
ML phylogeny of strain-specific consensus sequences for Tsu4 internal regions in all *S. paradoxus*, *S. cerevisiae*, *S. eubayanus*, and *S. uvarum*. Shown is the phylogeny reconstructed with 1,817 distinct alignment sites from 419 strain-specific consensus sequences. The consensus sequence is computed for each *S. paradoxus*, *S. cerevisiae*, *S. eubayanus*, and *S. uvarum* strain that has >0.75 depth and >0.9 breadth in its Tsu4 internal region. Tip points are colored by species. The phylogeny is midpoint rooted. Bootstrap supporting values are annotated for key nodes. Key clades are annotated with host lineage and/or clade numbers from the Tsu4 FLE phylogeny.

Using the short-read based WGS approach as above for *S. paradoxus* and *S. cerevisiae*, we first estimated Ty4/Tsu4 LTR and internal copy numbers in the context of host phylogenies for *S. eubayanus* (supplementary fig. S8, Supplementary Material online) and *S. uvarum* (supplementary fig. S9, Supplementary Material online), respectively. These results reveal that: (i) Tsu4 was present and Ty4 was absent in the ancestors of both *S. eubayanus* and *S. uvarum*; (ii) Tsu4 is broadly active in both species; and (ii) the Holarctic *S. eubayanus* lineage has the highest LTR copy number of Tsu4 in either species. We then computed consensus sequences for Tsu4 internal regions in all *S. paradoxus*, *S. cerevisiae*, *S. eubayanus*, and *S. uvarum* strains with an estimated copy number of >0.75 and generated a ML tree of strain-specific sequences across the combined dataset of four species (Fig. 5).

Key groupings in the strain-specific Tsu4 consensus tree (Fig. 5) agreed with the phylogeny of Tsu4 FLEs based on a smaller number of strains above (Fig. 4), validating both approaches. Notably, Tsu4 consensus sequences for all N. American *S. paradoxus* strains (together with the two *S. cerevisiae* Brazil 3 strains, UFMG-CM-Y641 and UFMG-CM-Y642) form a large monophyletic group (corresponding to Clade 3 in the FLE tree) that clusters most closely with consensus sequences from Mosaic 2 *S. cerevisiae* strains (corresponding to Clade 4). Likewise, the consensus sequence for the S. American *S. paradoxus* strain UFRJ50916 (corresponding to Clade 1) clusters with the S. American *S. cerevisiae* strain CQS (corresponding to Clade 2). All seven Holarctic *S. eubayanus* strains form a monophyletic group (corresponding to Clade 5) that clusters with *S. paradoxus* and *S. cerevisiae* consensus sequences and is distinct from Tsu4 sequences found in all other *S. eubayanus* or *S. uvarum* strains.

Phylogenetic analysis of strain-specific consensus sequences also revealed two other Tsu4 HTT events that could not be detected in the FLE phylogeny because of limited samples of WGAs. The first involves the two *S. cerevisiae* Brazil 3 strains (UFMG-CM-Y641 and UFMG-CM-Y642). Based on the location of Brazil 3 samples in the consensus sequence tree (Fig. 5) and the pattern of variation in the consensus sequences of all six *S. cerevisiae* strains that have Tsu4 (supplementary fig. S10, Supplementary Material online), we conclude that Tsu4 sequences in the *S. cerevisiae* Brazil 3 lineage arose from a HTT event that is distinct from those detected using FLEs in the *S. cerevisiae* Mosaic 2 or French Guiana lineages. The second involves the *S. eubayanus* Patagonia B strain yHAB565, whose Tsu4 consensus sequence is placed within the *S. uvarum* cluster. *Saccharomyces eubayanus* yHAB565 is placed correctly in the Patagonia B lineage in our host strain tree (supplementary fig. S8, Supplementary Material online), which rules out the possibility of sample mixups during sequencing or bioinformatic analysis and supports another HTT Tsu4 event from *S. uvarum* into *S. eubayanus*.

Taken together, our results suggest that the similarity between the Tsu4 clades in Holarctic *S. eubayanus*, *S. paradoxus*, and *S. cerevisiae* arose from parallel HTT events donated by an as-yet-unidentified *Saccharomyces* lineage. The substantial divergence between non-Holarctic *S. eubayanus* or *S. uvarum* and the clade comprised of Holarctic *S. eubayanus*, *S. paradoxus*, and *S. cerevisiae* suggests that this unknown donor is either an uncharacterized lineage of *S. eubayanus* or *S. uvarum* (e.g. West China *S. eubayanus* Bing et al. 2014) or potentially an undiscovered species related to *S. eubayanus* and *S. uvarum*. Additionally, we show that our novel strain-specific consensus sequence approach complements analysis of FLEs from WGAs and can reveal previously undetected cases of HTT in the abundant WGS datasets available for multiple yeast species.

### Tsu4 HTT Fuels the Evolution of Recombinant Clades in *S. mikatae* and *S. kudriavzevii*

Our phylogenetic network analysis of FLEs from the Ty4 family above revealed evidence of reticulation in *S. mikatae* Clade 11 and *S. kudriavzevii* Clade 12 (Fig. 4a) that could be caused by recombination between the Ty4 and Tsu4 subfamilies (Huson and Bryant 2006). To provide further evidence for recombination between the Ty4 and Tsu4 subfamilies, we first selected representative FLEs for pure Tsu4 (f32 from the *S. uvarum* Clade 8) and “pure” Ty4 (f49 from the *S. cerevisiae* Clade 13) from outgroup species not involved in the putative recombination events. We then plotted a sliding window of pairwise sequence divergence between these representative pure Ty4 and Tsu4 FLEs and a putatively pure *S. mikatae* Clade 10 FLE (f286 from IFO 1815) or a putatively recombinant *S. mikatae* Clade 11 FLE (f256 from IFO 1815) (supplementary fig. S11, Supplementary Material online). This analysis revealed that the 5′ internal region—including the complete *gag* gene and the first ∼500bp of *pol*—shows lower levels of divergence between *S. uvarum* Tsu4 and pure *S. mikatae* Clade 10 (supplementary fig. S11A, Supplementary Material online) than recombinant *S. mikatae* Clade 11 (supplementary fig. S11B, Supplementary Material online). Conversely, the same 5′ internal region shows higher levels of divergence between *S. cerevisiae* Ty4 and pure *S. mikatae* Clade 10 (supplementary fig. S11D, Supplementary Material online) than recombinant *S. mikatae* Clade 11 (supplementary fig. S11E, Supplementary Material online). These data indicate that the 5′ internal segment in Clade 11 is derived from the Ty4 subfamily, while the rest of the Clade 11 internal region is derived from the Tsu4 subfamily (Fig. 1).

**Fig. 6.**
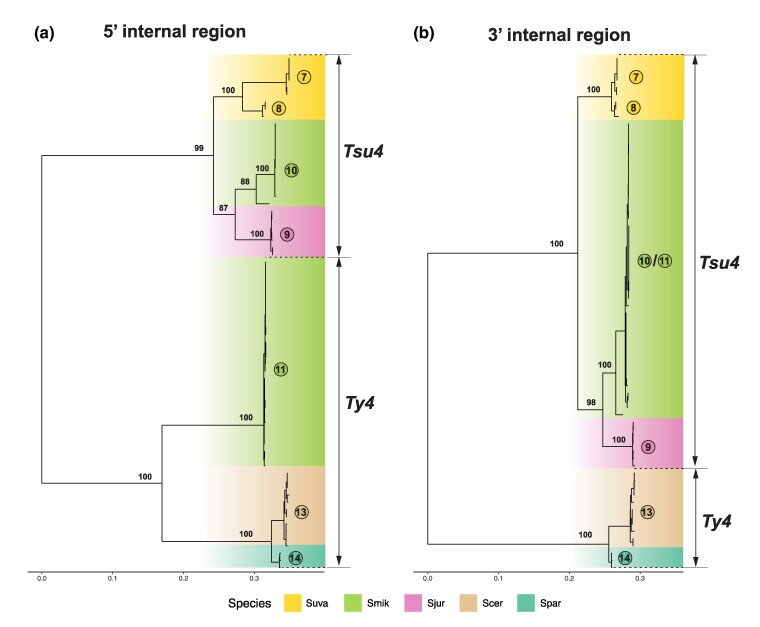
ML phylogeny for 5′ and 3′ internal regions from Tsu4 FLEs in *S. mikatae*, *S. jurei*, and *S. uvarum*, plus representatives of Ty4 elements. a) shows the ML phylogeny for 5′ internal region containing 459 distinct alignment sites; b) shows 3′ internal region containing 455 distinct alignment sites. Internal coding regions from 71 Ty4 and Tsu4 FLEs are included in both panels. Both trees are midpoint rooted, and visualized in the same tree scale which is shown in units of substitutions per site. Bootstrap supporting values are annotated for key nodes.

We then partitioned the multiple sequence alignment of Ty4 family FLE internal regions into 5′ and 3′ segments, and reconstructed ML phylogenies for both partitions from representative clades (Fig. 6). A striking discordance is detected in phylogenies reconstructed from 5′ and 3′ internal regions of Clade 11 sequences. In the 5′ partition, pure Clade 10 FLEs cluster with Tsu4 FLEs from *S. jurei* and *S. uvarum*, while recombinant Clade 11 FLEs cluster with strong support as a sister group to from *S. paradoxus*/*S. cerevisiae* in the Ty4 subfamily (Fig. 6a). In contrast, in the tree reconstructed from the 3′ partition *S. mikatae* Clades 10 and 11 form a single monophyletic group that is closely related to Tsu4 sequences from *S. jurei* (Fig. 6b).

Based on these results and presence of Ty4 solo LTRs in WGAs from *S. mikatae* and *S. jurei* (supplementary table S1, Supplementary Material online), we propose the following scenario for the evolution of *S. mikatae* Clades 10 and 11. A divergent Ty4 subfamily was previously active in an ancestor of *S. mikatae* and *S. jurei*, which has subsequently gone extinct in both species but left Ty4 internal sequences that were retained in the *S. mikatae* genome. A HTT event introduced the Tsu4 subfamily into the ancestor of *S. mikatae* and *S. jurei*, which evolved into Clade 10 in *S. mikatae* and Clade 9 in *S. jurei*. This Tsu4 HTT event explains the discordance between the Ty4 family and host species phylogenies previously reported for *S. mikatae* Bergman (2018). The donor for this Tsu4 HTT into the ancestor of *S. mikatae* and *S. jurei* is unknown but related to the ancestor to all extant Tsu4 FLEs in *S. eubayanus*, *S. uvarum*, *S. paradoxus* and *S. cerevisiae*. Recombination of the 5′ internal region from the now-extinct Ty4 subfamily in *S. mikatae* onto a Clade 10-like pure Tsu4 FLE created the “recombinant” Tsu4 Clade 11. This model explains the lack of Ty4 FLEs in *S. mikatae* and *S. jurei* (supplementary table S1, Supplementary Material online), the coexistence of two highly divergent clades in *S. mikatae* genomes (Fig. 4b), the long branch leading to Clade 11 in the FLE phylogeny (Fig. 4b), and reticulation between Ty4 and Tsu4 subfamilies for Clade 11 in the phylogenetic network (Fig. 4a).

**Fig. 7.**
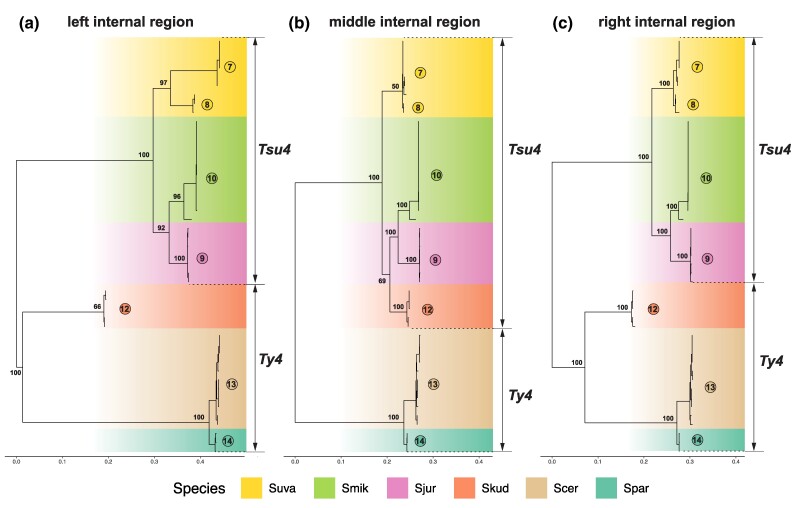
ML phylogeny for partitioned internal regions from recombinants in *S. kudriavzevii*, pure Tsu4 FLEs in *S. mikatae*, *S. jurei*, and *S. uvarum*, plus representatives of Ty4 elements. a) shows the ML phylogeny for left-side internal region based on 357 distinct alignment sites; b) for middle internal region based on 367 distinct alignment sites; c) for right-side internal region based on 423 distinct alignment sites. In all three panels, internal regions from 47 Ty4 and Tsu4 FLEs are included. All trees are midpoint rooted, and visualized in the same tree scale which is shown in units of substitutions per site. Bootstrap supporting values are annotated for key nodes.

We applied similar approaches to investigate whether reticulation in the phylogenetic network observed for *S. kudriavzevii* Clade 12 FLEs (Fig. 4a) also is caused by recombination between Ty4 and Tsu4 subfamilies. Sliding window analysis of a representative Clade 12 FLE versus pure Tsu4 and Ty4 from outgroup species revealed an ∼2 kb segment starting at the beginning of Pol that shows very high similarity to Tsu4 (supplementary fig. S11C, Supplementary Material online). Phylogenetic analysis of partitions corresponding to the “left,” “middle,” and “right” segments of FLE internal regions for representative clades reveals that the middle internal segment of Clade 12 is derived from the Tsu4 subfamily, while the left and right segments are divergent representatives of the Ty4 subfamily. Based on these results and presence of Ty4 LTRs in WGAs from *S. kudriavzevii* (supplementary table S1, Supplementary Material online), we propose that *S. kudriavzevii* ancestrally contained a divergent Ty4 subfamily which acquired a middle segment from a horizontally transferred Tsu4 by recombination. The Tsu4 clade that was horizontally transferred into *S. kudriavzevii* and the original pure *S. kudriavzevii* Ty4 clade have both subsequently gone extinct, leaving the recombinant Clade 12 as the only extant representative of the Ty4 family currently identified in *S. kudriavzevii*. Based on clustering of the middle internal region (Fig. 7b), the donor lineage for the Tsu4 HTT into *S. kudriavzevii* is related to the donor for Tsu4 HTT in *S. mikatae* and *S. jurei*. Together, the recombinant clades in *S. mikatae* and *S. kudriavzevii* support the conclusion that HTT mediates co-existence of Ty4 and Tsu4 subfamily sequences in the same genome which in turn provides substrate for recombination to generate new retrotransposon clades in *Saccharomyces*.

## Conclusions

Here, we addressed the impact of HTT on the evolution of the Ty4 family in *Saccharomyces* by integrating large-scale short-read WGS data and high-quality long-read WGAs from multiple *Saccharomyces* species. We showed that the previously detected Tsu4 HTT event in *S. paradoxus* (Bergman 2018) occurred in the ancestor of all American lineages and report new evidence for a second, more recent Tsu4 HTT in the South American lineage of *S. paradoxus*. We also showed that the previously reported presence of Tsu4 in *S. cerevisiae* (Bergman 2018; O’Donnell et al. 2023) is explained by independent HTT events into at least three *S. cerevisiae* in Central/South America lineages. One of the recipient lineages for Tsu4 (French Guiana) is also associated with HTT of another retrotransposon family (Ty1) and introgression of host genes from *S. paradoxus* (Peter et al. 2018; Bleykasten-Grosshans et al. 2021). The inference of multiple independent Tsu4 HTT events in both *S. paradoxus* and *S. cerevisiae* extends prior observations of parallel HTT events involving the Ty1 family in different lineages of *S. cerevisiae* (Czaja et al. 2020; Bleykasten-Grosshans et al. 2021). Together, these results suggest that, when conditions are favorable, parallel HTT events in different parts of a species range may be a common occurrence for *Saccharomyces* TE families. If so, the number of HTT events in *Saccharomyces* species cannot be reliably inferred from simple presence/absence data within species, and reconstructing the complex history of HTT events will require high-resolution phylogenetic data from large samples of FLEs or strain-specific consensus sequences. Similarly, the observation of parallel HTT events on short timescales for multiple yeast TE families suggests that large-scale surveys that detect HTT among distantly related taxa may underestimate the frequency of HTT in eukaryotic genome evolution (Peccoud et al. 2017, 2018).

Furthermore, we investigated the putative Tsu4 HTT event previously detected in *S. mikatae* (Bergman 2018) by generating new PacBio WGAs for two strains in this species (IFO 1815 and NBRC 10994), which revealed the presence of two active Ty4 family clades in *S. mikatae*. The first is a pure Tsu4 clade that we infer arose by a HTT event prior to the divergence of *S. mikatae* and *S. jurei*, which explains the previously reported discordance between Ty4 family and host species phylogenies for *S. mikatae* (Bergman 2018). We also found a second recombinant clade in *S. mikatae* that shares similarity to the Ty4 and Tsu4 subfamilies in different parts of the internal region. The recombinant Clade 11 implies the co-existence of internal sequences for both subfamilies in the *S. mikatae* genome at some point in history. Likewise, we identified a novel clade in *S. kudriavzevii* that similarly exhibits recombination between the Ty4 and Tsu4 subfamilies, and explains the divergent position of *S. kudriavzevii* FLEs in the Ty4 family phylogeny (Bergman 2018). The discovery of novel Ty4 family clades in *S. mikatae* and *S. kudriavzevii* that were likely generated by recombination between resident (ancestral) and horizontally transferred (derived) retrotransposon subfamilies generalizes similar results previously reported for the Ty1/Ty2 superfamily in *S. cerevisiae* (Jordan and McDonald 1998; Czaja et al. 2020; Bleykasten-Grosshans et al. 2021). Together these results suggest that recombination among divergent subfamilies that co-occur in the same species because of HTT may be a common mechanisms for the evolution of new TE lineages in *Saccharomyces* and possibly other organisms.

## Materials and Methods

### DNA Preparation, PacBio Sequencing and Genome Assembly of *S. mikatae* Strains IFO 1815 and NBRC 10994

To prepare DNA for PacBio sequencing, single colonies of strain IFO 1815 (NCYC 2888) and NBRC 10994 was inoculated in 7 ml yeast extract-peptone-dextrose (YPD) liquid broth and cultured for ∼24 hours at 30 °C. DNA was isolated using the Wizard genomic DNA purification kit (Promega), and a PacBio library was prepared using the SMRTbell Express template prep kit (Pacific Biosciences), following the >15-kb size-selection protocol that includes Covaris g-TUBE shearing. PacBio sequencing was performed using the Sequel II instrument (sequencing kit v2.1). Whole genome assemblies were generated by performing Flye (v2.9) (Kolmogorov et al. 2019) with parameters “–pacbio-raw -g 12 m.” Raw PacBio reads and genome assemblies of both *S. mikatae* strains have been submitted to NCBI under BioProject PRJNA934353.

### Genome Sequence Datasets

We compiled public short-read paired-end WGS datasets of multiple *Saccharomyces* species to generate intraspecific phylogenies and survey Ty4/Tsu4 subfamily content within species. WGS datasets for each *Saccharomyces* species were cleaned and normalized using the following steps: (i) raw reads with identical BioSample accession, strain name and sequencing strategy (i.e. sequencing instrument and layout) were merged according to the metatable provided by NCBI SRA; (ii) for the NCBI BioSample accessions that have multiple records after merging, only the record with the highest sequencing depth was retained; and (iii) all samples with sequencing depth <10× were removed. After these quality control processes, the integrated WGS dataset used in this study includes 2,404 *S. cerevisiae* strains (Skelly et al. 2013; Bergstrom et al. 2014; Almeida et al. 2015; Marsit et al. 2015; Song et al. 2015; Strope et al. 2015; Barbosa et al. 2016, 2018; Drozdova et al. 2016; Gallone et al. 2016; Gayevskiy et al. 2016; Goncalves et al. 2016; Zhu et al. 2016; Coi et al. 2017; Istace et al. 2017; Kita et al. 2017; Maclean et al. 2017; Yue et al. 2017; Duan et al. 2018; Legras et al. 2018; Peter et al. 2018; Fay et al. 2019; Kang et al. 2019; Ramazzotti et al. 2019; Basile et al. 2021; Bigey et al. 2021; Han et al. 2021), 370 *S. paradoxus* strains (Bergstrom et al. 2014; Leducq et al. 2016; Xia et al. 2017; Eberlein et al. 2019; Koufopanou et al. 2020; He et al. 2022; Peris et al. 2023), 18 *S. mikatae* strains (Peris et al. 2023), 62 *S. uvarum* strains (Scannell et al. 2011; Almeida et al. 2014; Macias et al. 2021; Peris et al. 2023), and 292 *S. eubayanus* strains (Peris et al. 2016; Brouwers et al. 2019; Salazar et al. 2019; Langdon et al. 2020; Nespolo et al. 2020; Bergin et al. 2022; Mardones et al. 2022; Molinet et al. 2022; Peris et al. 2023).

We complied public high-quality WGAs of *Saccharomyces* species to identify Ty4/Tsu4 copies and extract FLEs for phylogenetic analysis. These WGAs were generated mostly with long-read sequencing data (PacBio or ONT), however we also included three WGAs generated with short-read sequencing data for *S. cerevisiae* that showed evidence of Tsu4 internal regions (strain 245 from Marsit et al. 2015; strains AFQ and CDM from Peter et al. 2018) and two WGAs generated with short-read sequencing data for *S. arboricola* (strain H-6 from Liti et al. 2013 and strain ZP960 from Peris et al. 2023) that represent the best available WGAs for this species. In total, we analyzed 183 *S. cerevisiae* WGAs (Marsit et al. 2015; Yue et al. 2017; Peter et al. 2018; O’Donnell et al. 2023), 12 *S. paradoxus* WGAs (Yue et al. 2017; Eberlein et al. 2019; Chen et al. 2022b), two *S. mikatae* WGAs (this study), two *S. jurei* WGAs (Naseeb et al. 2018), three *S. kudriavzevii* WGAs (Boonekamp et al. 2018; Salzberg et al. 2022), two *S. arboricola* WGAs (Liti et al. 2013; Peris et al. 2023), two *S. uvarum* WGAs (Chen et al. 2022a; Salzberg et al. 2022), and four *S. eubayanus* WGAs (Brickwedde et al. 2018; Brouwers et al. 2019; Mardones et al. 2022).

### Ty4/Tsu4 Copy Number Estimates

Copy number of LTRs and internal regions for the Ty4 and Tsu4 subfamilies were estimated by the coverage module of McClintock 2 (Chen et al. 2023) using public short-read WGS datasets compiled above. For this analysis, we used McClintock revision 7aa5298 with parameters “--keep_intermediate minimal,coverage -m coverage.” Reference genomes used for WGS based copy number were as follows: *S. cerevisiae* laboratory strain S288c (UCSC version sacCer2); *S. paradoxus* European strain CBS432 (GCA_002079055.1) (Yue et al. 2017); *S. uvarum* European strain CBS 7001 (GCA_019953615.1) (Chen et al. 2022a); *S. eubayanus* Patagonia strain FM1318 (GCA_001298625.1) (Baker et al. 2015). The Ty query library used for this analysis is the same as in Czaja et al. (2020). The edge-trimming option in the McClintock coverage module was disabled by specifying “omit_edges” as “False” in the configuration file “config/coverage/coverage.py.” To reduce the influence of variable coverage and computing resources, samples with original fold-coverage greater than 100× were down-sampled to 100× using seqtk (v1.3) (https://github.com/lh3/seqtk).

### Phylogenetic Analysis of Host Species

For each species, multisample variant calling was performed with BCFtools (v1.16, “bcftools mpileup -a ‘FORMAT/DP’ -Q 20 -q 20”; “bcftools call -f GQ,GP -mv –skip-variants indels”) (Li 2011) using BAM files generated by McClintock 2 (Chen et al. 2023). Alignments with mapping quality less than 20, or bases with quality score less than 20, were removed. All indels were excluded from variant calling. Subsequently, the SNP matrix was filtered with BCFtools filter (v1.16) to discard sites with polymorphic probabilities under 99%; or genotypes with average supporting read depth less than 10×. Vcf2phylip (revision 0eb1b80) (https://github.com/edgardomortiz/vcf2phylip/tree/v2.0) was executed to create multisequence alignments from the filtered VCF file. For all species other than *S. cerevisiae*, maximum likelihood (ML) phylogenetic analysis was performed with RAxML (v8.2.12, “-f a -x 23333 -p 2333 –no-bfgs”) (Stamatakis 2014) applying GTRGAMMA model and 100 times of bootstrap resampling. The host species trees were mid-point rooted and visualized in R (v4.2.3) using packages phytools (v1.5_1) (Revell 2012) and ggtree (v3.6.0) (Yu et al. 2017), respectively. For *S. cerevisiae*, we followed the workflow in Peter et al. (2018): “snpgdsVCF2GDS” and “snpgdsDiss” from package SNPRelate (v1.32.0) (Zheng et al. 2012) were used to create the distance matrix from SNP data, and then function “bionj” from ape (v5.7_1) (Paradis et al. 2004) was used to reconstruct the neighbor joining (NJ) tree.

### Annotation and Sequence Analysis of Full-Length Elements from Whole Genome Assemblies

Ty elements were annotated in WGAs using a RepeatMasker-based pipeline previously described in Czaja et al. (2020) updated to use RepeatMasker v4.0.9. Three *S. cerevisiae* Ty4 elements with secondary FLE insertions from other Ty families were excluded from the final dataset to prevent multisequence alignment artifacts. De novo LTR element prediction was performed using LTRharvest (“-seed 100 -minlenltr 100 -maxlenltr 1000 -mindistltr 1500 -maxdistltr 15000 -similar 80.0 -xdrop 5 -mat 2 -mis -2 -ins -3 -del -3 -mintsd 5 -maxtsd 5 -motif tgca -motifmis 0 -vic 60 -overlaps best”) followed by LTRdigest (PFAM models: PF00078, PF00665, PF01021, PF03732, PF07727, PF12384, PF13976) in GenomeTools 1.6.1 (Ellinghaus et al. 2008; Steinbiss et al. 2009; Gremme et al. 2013; Mistry et al. 2021). Multisequence alignments of annotated FLEs were generated using MAFFT (v7.508) with default parameters (Katoh and Standley 2013). Sub-regions of FLEs in alignments were identified by aligning the “Tsu4p_nw” sequence from a public database of annotated canonical yeast transposons (https://github.com/bergmanlab/yeast-transposons) with the FLE dataset, then selecting sub-regions with seqkit subseq (v0.16.1) (Shen et al. 2016). ML phylogenetic trees were reconstructed using RAxML (v8.2.12, “-f a -x 23333 -p 2333 –no-bfgs”) (Stamatakis 2014) with GTRGAMMA model and 100 bootstrap replicates. Phylogenetic network analysis was performed with SplitsTree4 (v4.15.1) (Huson and Bryant 2006) applying the “Uncorrect_P” model and “NeighborNet” method. Pairwise sequence divergence was calculated based on Kimura’s 2-parameter substitution model with 50-bp sliding window size and 10-bp step size with R package spider (GitHub revision e93c5b4) (Brown et al. 2012) and phangorn (v2.11.1) (Schliep 2011) in R (v4.2.3).

### Phylogenetic Analysis of Tsu4 Strain-Specific Consensus Sequences

Strain-specific consensus sequences were generated with BCFtools (v1.16, “bcftools mpileup -a ‘FORMAT/DP’ -Q 20 -q 20”; “bcftools call -f GQ,GP -mv –skip-variants indels; bcftools consensus”) (Li 2011) using BAM files previously generated by McClintock 2 (Chen et al. 2023). The percentage of bases supported by mapped reads (i.e. breadth) was calculated with BEDtools (v2.30.0, “bedtools genomecov -d -split”) (Quinlan and Hall 2010). To avoid generating consensus sequences that are biased towards the Tsu4 reference sequence, samples with normalized Tsu4 depth less than 0.75 (estimated by McClintock coverage module) or breadth less than 0.9 (estimated by BEDtools genomecov) were removed from consensus sequence analysis. A multisequence alignment of strain-specific consensus sequences was generated using MAFFT (v7.508) (Katoh and Standley 2013) with default parameters. ML phylogenetic analysis was performed using RAxML (v8.2.12, “-f a -x 23333 -p 2333 –no-bfgs”) applying GTRGAMMA model and 100 bootstrap replicates (Stamatakis 2014). The ML tree was mid-point rooted using R package phytools (v1.2_0) (Revell 2012) and then visualized using ggtree (v3.6.0) (Yu et al. 2017).

## Supplementary Material

evaf004_Supplementary_Data

## Data Availability

New sequence data reported in this article are available at the National Center for Biotechnology Information under accession PRJNA934353. All other data underlying this article are available in the article and in its online supplementary material.

## References

[evaf004-B1] Albertin W, Chernova M, Durrens P, Guichoux E, Sherman DJ, Masneuf-Pomarede I, Marullo P. Many interspecific chromosomal introgressions are highly prevalent in Holarctic *Saccharomyces uvarum* strains found in human-related fermentations. Yeast. 2018:35(1):141–156. 10.1002/yea.v35.1. 28779574

[evaf004-B2] Almeida P, Barbosa R, Zalar P, Imanishi Y, Shimizu K, Turchetti B, Legras J-L, Serra M, Dequin S, Couloux A, et al A population genomics insight into the Mediterranean origins of wine yeast domestication. Mol Ecol. 2015:24(21):5412–5427. 10.1111/mec.2015.24.issue-21. 26248006

[evaf004-B3] Almeida P, Goncalves C, Teixeira S, Libkind D, Bontrager M, Masneuf-Pomarede I, Albertin W, Durrens P, Sherman DJ, Marullo P, et al A Gondwanan imprint on global diversity and domestication of wine and cider yeast *Saccharomyces uvarum*. Nat Commun. 2014:5(1):4044. 10.1038/ncomms5044. 24887054 PMC5081218

[evaf004-B4] Amyotte SG, Tan X, Pennerman K, del Mar Jimenez-Gasco M, Klosterman SJ, Ma L-J, Dobinson KF, Veronese P. Transposable elements in phytopathogenic Verticillium spp.: insights into genome evolution and inter- and intra-specific diversification. BMC Genomics. 2012:13(1):314. 10.1186/1471-2164-13-314. 22800085 PMC3441728

[evaf004-B5] Baker E, Wang B, Bellora N, Peris D, Hulfachor AB, Koshalek JA, Adams M, Libkind D, Hittinger CT. The genome sequence of *Saccharomyces eubayanus* and the domestication of lager-brewing yeasts. Mol Biol Evol. 2015:32(11):2818–2831. 10.1093/molbev/msv168. 26269586 PMC4651232

[evaf004-B6] Barbosa R, Almeida P, Safar SVB, Santos RO, Morais PB, Nielly-Thibault L, Leducq J-B, Landry CR, Goncalves P, Rosa CA, et al Evidence of natural hybridization in Brazilian wild lineages of *Saccharomyces cerevisiae*. Genome Biol Evol. 2016:8(2):317–329. 10.1093/gbe/evv263. 26782936 PMC4779607

[evaf004-B7] Barbosa R, Pontes A, Santos RO, Montandon GG, de Ponzzes-Gomes CM, Morais PB, Gonçalves P, Rosa CA, Sampaio JP. Multiple rounds of artificial selection promote microbe secondary domestication—the case of cachaça yeasts. Genome Biol Evol. 2018:10(8):1939–1955. 10.1093/gbe/evy132. 29982460 PMC6101510

[evaf004-B8] Basile A, De Pascale F, Bianca F, Rossi A, Frizzarin M, De Bernardini N, Bosaro M, Baldisseri A, Antoniali P, Lopreiato R, et al Large-scale sequencing and comparative analysis of oenological *Saccharomyces cerevisiae* strains supported by nanopore refinement of key genomes. Food Microbiol. 2021:97:103753. 10.1016/j.fm.2021.103753. 33653526

[evaf004-B9] Bergin SA, Allen S, Hession C, Ó Cinnéide E, Ryan A, Byrne KP, Ó Cróinín T, Wolfe KH, Butler G. Identification of European isolates of the lager yeast parent *Saccharomyces eubayanus*. FEMS Yeast Res. 2022:22(1):foac053. 10.1093/femsyr/foac053. 36473696 PMC9726447

[evaf004-B10] Bergman CM . Horizontal transfer and proliferation of Tsu4 in *Saccharomyces paradoxus*. Mob DNA. 2018:9(1):18. 10.1186/s13100-018-0122-7. 29942366 PMC5998506

[evaf004-B11] Bergstrom A, Simpson JT, Salinas F, Barre B, Parts L, Zia A, Ba N, N A, Moses AM, Louis EJ, et al A high-definition view of functional genetic variation from natural yeast genomes. Mol Biol Evol. 2014:31(4):872–888. 10.1093/molbev/msu037. 24425782 PMC3969562

[evaf004-B12] Bigey F, Segond D, Friedrich A, Guezenec S, Bourgais A, Huyghe L, Agier N, Nidelet T, Sicard D. Evidence for two main domestication trajectories in *Saccharomyces cerevisiae* linked to distinct bread-making processes. Curr Biol. 2021:31(4):722–732.e5. 10.1016/j.cub.2020.11.016. 33301710

[evaf004-B13] Bing J, Han P-J, Liu W-Q, Wang Q-M, Bai F-Y. Evidence for a Far East Asian origin of lager beer yeast. Curr Biol. 2014:24(10):R380–R381. 10.1016/j.cub.2014.04.031. 24845661

[evaf004-B14] Bleykasten-Grosshans C, Fabrizio R, Friedrich A, Schacherer J. Species-wide transposable element repertoires retrace the evolutionary history of the *Saccharomyces cerevisiae* host. Mol Biol Evol. 2021:38:4334–4345. 10.1093/molbev/msab171. 34115140 PMC8476168

[evaf004-B15] Boonekamp FJ, Dashko S, van den Broek M, Gehrmann T, Daran J-M, Daran-Lapujade P. The genetic makeup and expression of the glycolytic and fermentative pathways are highly conserved within the Saccharomyces genus. Front Genet. 2018:9:504. 10.3389/fgene.2018.00504. 30505317 PMC6250768

[evaf004-B16] Borneman AR, Pretorius IS. Genomic insights into the Saccharomyces sensu stricto complex. Genetics. 2015:199:281–291. 10.1534/genetics.114.173633. 25657346 PMC4317643

[evaf004-B17] Boynton PJ, Greig D. The ecology and evolution of non-domesticated Saccharomyces species. Yeast. 2014:31:449–462. 10.1002/yea.3040. 25242436 PMC4282469

[evaf004-B18] Brickwedde A, Brouwers N, van den Broek M, Gallego Murillo JS, Fraiture JL, Pronk JT, Daran J-M. Structural, physiological and regulatory analysis of maltose transporter genes in *Saccharomyces eubayanus* CBS 12357T. Front Microbiol. 2018:9:1786. 10.3389/fmicb.2018.01786. 30147677 PMC6097016

[evaf004-B19] Brouwers N, Brickwedde A, Gorter de Vries AR, van den Broek M, Weening SM, den Eijnden Lvan, Diderich JA, Bai F-Y, Pronk JT, G. Daran J-M. Himalayan *Saccharomyces eubayanus* genome sequences reveal genetic markers explaining heterotic maltotriose consumption by *Saccharomyces pastorianus* hybrids. Appl Environ Microbiol. 2019:85(22):e01516–19. 10.1128/AEM.01516-19. 31519660 PMC6821976

[evaf004-B20] Brown SDJ, Collins RA, Boyer S, Lefort M-C, Malumbres-Olarte J, Vink CJ, Cruickshank RH. Spider: an R package for the analysis of species identity and evolution, with particular reference to DNA barcoding. Mol Ecol Resour. 2012:12(3):562–565. 10.1111/men.2012.12.issue-3. 22243808

[evaf004-B21] Carr M, Bensasson D, Bergman CM. Evolutionary genomics of transposable elements in *Saccharomyces cerevisiae*. PLoS One. 2012:7:e50978. 10.1371/journal.pone.0050978. 23226439 PMC3511429

[evaf004-B22] Chen J, Basting PJ, Han S, Garfinkel DJ, Bergman CM. Reproducible evaluation of transposable element detectors with McClintock 2 guides accurate inference of Ty insertion patterns in yeast. Mob DNA. 2023:14:8. 10.1186/s13100-023-00296-4. 37452430 PMC10347736

[evaf004-B23] Chen J, Garfinkel DJ, Bergman CM. Long-read genome assembly of *Saccharomyces uvarum* strain CBS 7001. Microbiol Resour Announc. 2022a:11:e00972–21. 10.1128/mra.00972-21. 34989601 PMC8759399

[evaf004-B24] Chen J, McQueary H, Hall DW, Philippsen P, Garfinkel DJ, Bergman CM. Genome assembly of the Ty1-less *Saccharomyces paradoxus* strain DG1768. Microbiol Resour Announc. 2022b:11:e0086821. 10.1128/mra.00868-21. 35049349 PMC8772602

[evaf004-B25] Cliften P, Sudarsanam P, Desikan A, Fulton L, Fulton B, Majors J, Waterston R, Cohen BA, Johnston M. Finding functional features in Saccharomyces genomes by phylogenetic footprinting. Science. 2003:301:71–76. 10.1126/science.1084337. 12775844

[evaf004-B26] Coi AL, Bigey F, Mallet S, Marsit S, Zara G, Gladieux P, Galeote V, Budroni M, Dequin S, Legras JL. Genomic signatures of adaptation to wine biological ageing conditions in biofilm-forming flor yeasts. Mol Ecol. 2017:26:2150–2166. 10.1111/mec.2017.26.issue-7. 28192619

[evaf004-B27] Czaja W, Bensasson D, Ahn HW, Garfinkel DJ, Bergman CM. Evolution of Ty1 copy number control in yeast by horizontal transfer and recombination. PLoS Genet. 2020:16(2):e1008632. 10.1371/journal.pgen.1008632. 32084126 PMC7055915

[evaf004-B28] Daboussi M-J, Daviere J-M, Graziani S, Langin T. Evolution of the Fot1 transposons in the genus Fusarium: discontinuous distribution and epigenetic inactivation. Mol Biol Evol. 2002:19(4):510–520. 10.1093/oxfordjournals.molbev.a004106. 11919292

[evaf004-B29] David KT, Harrison M-C, Opulente DA, LaBella AL, Wolters JF, Zhou X, Shen X-X, Groenewald M, Pennell M, Hittinger CT, et al Saccharomycotina yeasts defy long-standing macroecological patterns. Proc Natl Acad Sci U S A. 2024:121(10):e2316031121. 10.1073/pnas.2316031121. 38412132 PMC10927492

[evaf004-B30] Dobinson KF, Harris RE, Hamer JE. Grasshopper, a long terminal repeat (LTR) retroelement in the phytopathogenic fungus *Magnaporthe grisea*. Mol Plant Microbe Interact. 1993:6(1):114–126. 10.1094/MPMI-6-114. 7679935

[evaf004-B31] Dotto BR, Carvalho EL, Silva AF, Silva D, Fernando L, Pinto PM, Ortiz MF, Wallau GL. HTT-DB: horizontally transferred transposable elements database. Bioinformatics. 2015:31(17):2915–2917. 10.1093/bioinformatics/btv281. 25940562

[evaf004-B32] Drozdova PB, Tarasov OV, Matveenko AG, Radchenko EA, Sopova JV, Polev DE, Inge-Vechtomov SG, Dobrynin PV. Genome sequencing and comparative analysis of *Saccharomyces cerevisiae* strains of the Peterhof genetic collection. PLoS One. 2016:11:e0154722. 10.1371/journal.pone.0154722. 27152522 PMC4859572

[evaf004-B33] Duan S-F, Han P-J, Wang Q-M, Liu W-Q, Shi J-Y, Li K, Zhang X-L, Bai F-Y. The origin and adaptive evolution of domesticated populations of yeast from Far East Asia. Nat Commun. 2018:9:2690. 10.1038/s41467-018-05106-7. 30002370 PMC6043522

[evaf004-B34] Eberlein C, Henault M, Fijarczyk A, Charron G, Bouvier M, Kohn LM, Anderson JB, Landry CR. Hybridization is a recurrent evolutionary stimulus in wild yeast speciation. Nat Commun. 2019:10:923. 10.1038/s41467-019-08809-7. 30804385 PMC6389940

[evaf004-B35] Ellinghaus D, Kurtz S, Willhoeft U. LTRharvest, an efficient and flexible software for de novo detection of LTR retrotransposons. BMC Bioinformatics. 2008:9:18. 10.1186/1471-2105-9-18. 18194517 PMC2253517

[evaf004-B36] Farabaugh PJ, Fink GR. Insertion of the eukaryotic transposable element Ty1 creates a 5-base pair duplication. Nature. 1980:286:352–356. 10.1038/286352a0. 6250062

[evaf004-B37] Fay JC, Liu P, Ong GT, Dunham MJ, Cromie GA, Jeffery EW, Ludlow CL, Dudley AM. A polyploid admixed origin of beer yeasts derived from European and Asian wine populations. PLoS Biol. 2019:17:e3000147. 10.1371/journal.pbio.3000147. 30835725 PMC6400334

[evaf004-B38] Gallone B, Steensels J, Prahl T, Soriaga L, Saels V, Herrera-Malaver B, Merlevede A, Roncoroni M, Voordeckers K, Miraglia L, et al Domestication and divergence of *Saccharomyces cerevisiae* beer yeasts. Cell. 2016:166:1397–1410.e16. 10.1016/j.cell.2016.08.020. 27610566 PMC5018251

[evaf004-B39] Gayevskiy V, Lee S, Goddard MR. European derived *Saccharomyces cerevisiae* colonisation of New Zealand vineyards aided by humans. FEMS Yeast Res. 2016:16:fow091. 10.1093/femsyr/fow091. 27744274 PMC5094284

[evaf004-B40] Gilbert C, Feschotte C. Horizontal acquisition of transposable elements and viral sequences: patterns and consequences. Curr Opin Genetics Dev. 2018:49:15–24. 10.1016/j.gde.2018.02.007. PMC6069605 29505963

[evaf004-B41] Goncalves M, Pontes A, Almeida P, Barbosa R, Serra M, Libkind D, Hutzler M, Goncalves P, Sampaio J. Distinct domestication trajectories in top-fermenting beer yeasts and wine yeasts. Curr Biol. 2016:26:2750–2761. 10.1016/j.cub.2016.08.040. 27720622

[evaf004-B42] Gremme G, Steinbiss S, Kurtz S. GenomeTools: a comprehensive software library for efficient processing of structured genome annotations. IEEE/ACM Trans Comput Biol Bioinform. 2013:10:645–656. 10.1109/TCBB.2013.68. 24091398

[evaf004-B43] Han D-Y, Han P-J, Rumbold K, Koricha AD, Duan S-F, Song L, Shi J-Y, Li K, Wang Q-M, Bai F-Y. Adaptive gene content and allele distribution variations in the wild and domesticated populations of *Saccharomyces cerevisiae*. Front Microbiol. 2021:12:631250. 10.3389/fmicb.2021.631250. 33679656 PMC7925643

[evaf004-B44] Hannon-Hatfield JA, Chen J, Bergman CM, Garfinkel DJ. Evolution of a restriction factor by domestication of a yeast retrotransposon. Mol Biol Evol. 2024:41:msae050. 10.1093/molbev/msae050. 38442736 PMC10951436

[evaf004-B45] He P-Y, Shao X-Q, Duan S-F, Han D-Y, Li K, Shi J-Y, Zhang R-P, Han P-J, Wang Q-M, Bai F-Y. Highly diverged lineages of *Saccharomyces paradoxus* in temperate to subtropical climate zones in China. Yeast. 2022:39(1-2):69–82. 10.1002/yea.v39.1-2. 34961959

[evaf004-B46] Henault M, Eberlein C, Charron G, Durand E, Nielly-Thibault L, Martin H, Landry CR. Yeast population genomics goes wild: the case of *Saccharomyces paradoxus*. In: Polz MF, Rajora OP, editors. Population genomics: microorganisms. Cham: Springer International Publishing; 2019. p. 207–230.

[evaf004-B47] Henault M, Marsit S, Charron G, Landry CR. The effect of hybridization on transposable element accumulation in an undomesticated fungal species. Elife. 2020:9:e60474. 10.7554/eLife.60474. 32955438 PMC7584455

[evaf004-B48] Huson DH, Bryant D. Application of phylogenetic networks in evolutionary studies. Mol Biol Evol. 2006:23(2):254–267. 10.1093/molbev/msj030. 16221896

[evaf004-B49] Hyma KE, Fay JC. Mixing of vineyard and oak-tree ecotypes of *Saccharomyces cerevisiae* in North American vineyards. Mol Ecol. 2013:22(11):2917–2930. 10.1111/mec.2013.22.issue-11. 23286354 PMC3620907

[evaf004-B50] Istace B, Friedrich A, d’Agata L, Faye S, Payen E, Beluche O, Caradec C, Davidas S, Cruaud C, Liti G, et al De novo assembly and population genomic survey of natural yeast isolates with the Oxford Nanopore MinION sequencer. Gigascience. 2017:6:1–13. 10.1093/gigascience/giw018. PMC5466710 28369459

[evaf004-B51] Janetzky B, Lehle L. Ty4, a new retrotransposon from *Saccharomyces cerevisiae*, flanked by tau-elements. J Biol Chem. 1992:267:19798–19805. 10.1016/S0021-9258(19)88624-6. 1328182

[evaf004-B52] Jordan IK, McDonald JF. Evidence for the role of recombination in the regulatory evolution of *Saccharomyces cerevisiae* Ty elements. J Mol Evol. 1998:47:14–20. 10.1007/PL00006358. 9664692

[evaf004-B53] Kang K, Bergdahl B, Machado D, Dato L, Han T-L, Li J, Villas-Boas S, Herrgard MJ, Forster J, Panagiotou G. Linking genetic, metabolic, and phenotypic diversity among *Saccharomyces cerevisiae* strains using multi-omics associations. Gigascience. 2019:8:giz015. 10.1093/gigascience/giz015. 30715293 PMC6446221

[evaf004-B54] Katoh K, Standley DM. MAFFT multiple sequence alignment software version 7: improvements in performance and usability. Mol Biol Evol. 2013:30:772–780. 10.1093/molbev/mst010. 23329690 PMC3603318

[evaf004-B55] Kita R, Venkataram S, Zhou Y, Fraser HB. High-resolution mapping of cis-regulatory variation in budding yeast. Proc Natl Acad Sci U S A. 2017:114:E10736–E10744. 10.1073/pnas.1717421114. 29183975 PMC5740631

[evaf004-B56] Kolmogorov M, Yuan J, Lin Y, Pevzner PA. Assembly of long, error-prone reads using repeat graphs. Nat Biotechnol. 2019:37:540–546. 10.1038/s41587-019-0072-8. 30936562

[evaf004-B57] Koufopanou V, Hughes J, Bell G, Burt A. The spatial scale of genetic differentiation in a model organism: the wild yeast *Saccharomyces paradoxus*. Philos Trans R Soc Lond B Biol Sci. 2006:361:1941–1946. 10.1098/rstb.2006.1922. 17028086 PMC1764930

[evaf004-B58] Koufopanou V, Lomas S, Pronina O, Almeida P, Sampaio JP, Mousseau T, Liti G, Burt A. Population size, sex and purifying selection: comparative genomics of two sister taxa of the wild yeast *Saccharomyces paradoxus*. Genome Biol Evol. 2020:12:1636–1645. 10.1093/gbe/evaa141. 33011797 PMC7533043

[evaf004-B59] Kuehne HA, Murphy HA, Francis C, Sniegowski PD. Allopatric divergence, secondary contact, and genetic isolation in wild yeast populations. Curr Biol. 2007:17:407–411. 10.1016/j.cub.2006.12.047. 17306538

[evaf004-B60] Langdon QK, Peris D, Baker EP, Opulente DA, Nguyen H-V, Bond U, Gonçalves P, Sampaio JP, Libkind D, Hittinger CT. Fermentation innovation through complex hybridization of wild and domesticated yeasts. Nat Ecol Evol. 2019:3(11):1576–1586. 10.1038/s41559-019-0998-8. 31636426 PMC7295394

[evaf004-B61] Langdon QK, Peris D, Eizaguirre JI, Opulente DA, Buh KV, Sylvester K, Jarzyna M, Rodríguez ME, Lopes CA, Libkind D, et al Postglacial migration shaped the genomic diversity and global distribution of the wild ancestor of lager-brewing hybrids. PLoS Genet. 2020:16(4):e1008680. 10.1371/journal.pgen.1008680. 32251477 PMC7162524

[evaf004-B62] Leducq J-B, Charron G, Samani P, Dubé AK, Sylvester K, James B, Almeida P, Sampaio JP, Hittinger CT, Bell G, et al Local climatic adaptation in a widespread microorganism. Proc R Soc Lond B Biol Sci. 2014:281(1777):20132472. 10.1098/rspb.2013.2472. PMC3896012 24403328

[evaf004-B63] Leducq J-B, Nielly-Thibault L, Charron G, Eberlein C, Verta J-P, Samani P, Sylvester K, Hittinger CT, Bell G, Landry CR. Speciation driven by hybridization and chromosomal plasticity in a wild yeast. Nat Microbiol. 2016:1:15003. 10.1038/nmicrobiol.2015.3. 27571751

[evaf004-B64] Legras J-L, Galeote V, Bigey F, Camarasa C, Marsit S, Nidelet T, Sanchez I, Couloux A, Guy J, Franco-Duarte R, et al Adaptation of *S. cerevisiae* to fermented food environments reveals remarkable genome plasticity and the footprints of domestication. Mol Biol Evol. 2018:35(7):1712–1727. 10.1093/molbev/msy066. 29746697 PMC5995190

[evaf004-B65] Li H . A statistical framework for SNP calling, mutation discovery, association mapping and population genetical parameter estimation from sequencing data. Bioinformatics. 2011:27(21):2987–2993. 10.1093/bioinformatics/btr509. 21903627 PMC3198575

[evaf004-B66] Libkind D, Hittinger CT, Valerio E, Goncalves C, Dover J, Johnston M, Goncalves P, Sampaio JP. Microbe domestication and the identification of the wild genetic stock of lager-brewing yeast. Proc Natl Acad Sci U S A. 2011:108:14539–14544. 10.1073/pnas.1105430108. 21873232 PMC3167505

[evaf004-B67] Liti G, Ba ANN, Blythe M, Müller CA, Bergström A, Cubillos FA, Dafhnis-Calas F, Khoshraftar S, Malla S, Mehta N, et al High quality de novo sequencing and assembly of the *Saccharomyces arboricolus* genome. BMC Genomics. 2013:14:69. 10.1186/1471-2164-14-69. 23368932 PMC3599269

[evaf004-B68] Liti G, Barton DBH, Louis EJ. Sequence diversity, reproductive isolation and species concepts in Saccharomyces. Genetics. 2006:174:839–850. 10.1534/genetics.106.062166. 16951060 PMC1602076

[evaf004-B69] Liti G, Carter DM, Moses AM, Warringer J, Parts L, James SA, Davey RP, Roberts IN, Burt A, Koufopanou V, et al Population genomics of domestic and wild yeasts. Nature. 2009:458:337–341. 10.1038/nature07743. 19212322 PMC2659681

[evaf004-B70] Liti G, Peruffo A, James SA, Roberts IN, Louis EJ. Inferences of evolutionary relationships from a population survey of LTR-retrotransposons and telomeric-associated sequences in the Saccharomyces sensu stricto complex. Yeast. 2005:22:177–192. 10.1002/(ISSN)1097-0061. 15704235

[evaf004-B71] Macias LG, Flores MG, Adam AC, Rodriguez ME, Querol A, Barrio E, Lopes CA, Perez-Torrado R. Convergent adaptation of Saccharomyces uvarum to sulfite, an antimicrobial preservative widely used in human-driven fermentations. PLoS Genet. 2021:17:e1009872. 10.1371/journal.pgen.1009872. 34762651 PMC8631656

[evaf004-B72] Maclean CJ, Metzger BPH, Yang J-R, Ho W-C, Moyers B, Zhang J. Deciphering the genic basis of yeast fitness variation by simultaneous forward and reverse genetics. Mol Biol Evol. 2017:34:2486–2502. 10.1093/molbev/msx151. 28472365 PMC12104513

[evaf004-B73] Mardones W, Villarroel CA, Abarca V, Urbina K, Peña TA, Molinet J, Nespolo RF, Cubillos FA. Rapid selection response to ethanol in *Saccharomyces eubayanus* emulates the domestication process under brewing conditions. Microb Biotechnol. 2022:15:967–984. 10.1111/mbt2.v15.3. 33755311 PMC8913853

[evaf004-B74] Marsit S, Mena A, Bigey F, Sauvage F-X, Couloux A, Guy J, Legras J-L, Barrio E, Dequin S, Galeote V. Evolutionary advantage conferred by an eukaryote-to-eukaryote gene transfer event in wine yeasts. Mol Biol Evol. 2015:32:1695–1707. 10.1093/molbev/msv057. 25750179 PMC4476156

[evaf004-B75] Mistry J, Chuguransky S, Williams L, Qureshi M, Salazar G, Sonnhammer ELL, Tosatto SCE, Paladin L, Raj S, Richardson LJ, et al Pfam: the protein families database in 2021. Nucleic Acids Res. 2021:49:D412–D419. 10.1093/nar/gkaa913. 33125078 PMC7779014

[evaf004-B76] Molinet J, Urbina K, Villegas C, Abarca V, Oporto CI, Villarreal P, Villarroel CA, Salinas F, Nespolo RF, Cubillos FA. A *Saccharomyces eubayanus* haploid resource for research studies. Sci Rep. 2022:12:5976. 10.1038/s41598-022-10048-8. 35396494 PMC8993842

[evaf004-B77] Moore SP, Liti G, Stefanisko KM, Nyswaner KM, Chang C, Louis EJ, Garfinkel DJ. Analysis of a Ty1-less variant of *Saccharomyces paradoxus*: the gain and loss of Ty1 elements. Yeast. 2004:21(8):649–660. 10.1002/yea.v21:8. 15197730

[evaf004-B78] Myers EW, Sutton GG, Delcher AL, Dew IM, Fasulo DP, Flanigan MJ, Kravitz SA, Mobarry CM, Reinert KH, Remington KA, et al A whole-genome assembly of Drosophila. Science. 2000:287:2196–2204. 10.1126/science.287.5461.2196. 10731133

[evaf004-B79] Naseeb S, Alsammar H, Burgis T, Donaldson I, Knyazev N, Knight C, Delneri D. Whole genome sequencing, de novo assembly and phenotypic profiling for the new budding yeast species *Saccharomyces jurei*. G3 (Bethesda). 2018:8(9):2967–2977. 10.1534/g3.118.200476. 30097472 PMC6118302

[evaf004-B80] Naumov GI, James SA, Naumova ES, Louis EJ, Roberts IN. Three new species in the Saccharomyces sensu stricto complex: *Saccharomyces cariocanus*, *Saccharomyces kudriavzevii* and *Saccharomyces mikatae*. Int J Syst Evol Microbiol. 2000:50(5):1931–1942. 10.1099/00207713-50-5-1931. 11034507

[evaf004-B81] Naumov GI, Naumova ES, Sniegowski PD. Differentiation of European and Far East Asian populations of *Saccharomyces paradoxus* by allozyme analysis. Int J Syst Bacteriol. 1997:47:341–344. 10.1099/00207713-47-2-341. 9103619

[evaf004-B82] Nespolo RF, Villarroel CA, Oporto CI, Tapia SM, Vega-Macaya F, Urbina K, Chiara MD, Mozzachiodi S, Mikhalev E, Thompson D, et al An out-of-patagonia migration explains the worldwide diversity and distribution of *Saccharomyces eubayanus* lineages. PLoS Genet. 2020:16:e1008777. 10.1371/journal.pgen.1008777. 32357148 PMC7219788

[evaf004-B83] Neuveglise C, Feldmann H, Bon E, Gaillardin C, Casaregola S. Genomic evolution of the long terminal repeat retrotransposons in hemiascomycetous yeasts. Genome Res. 2002:12:930–943. 10.1101/gr.219202. 12045146 PMC1383729

[evaf004-B84] Novikova O, Fet V, Blinov A. Non-LTR retrotransposons in fungi. Funct Integr Genomics. 2009:9:27–42. 10.1007/s10142-008-0093-8. 18677522

[evaf004-B85] O’Donnell S, Yue J-X, Saada OA, Agier N, Caradec C, Cokelaer T, De Chiara M, Delmas S, Dutreux F, Fournier T, et al Telomere-to-telomere assemblies of 142 strains characterize the genome structural landscape in *Saccharomyces cerevisiae*. Nat Genet. 2023:55:1390–1399. 10.1038/s41588-023-01459-y. 37524789 PMC10412453

[evaf004-B86] Okuno M, Kajitani R, Ryusui R, Morimoto H, Kodama Y, Itoh T. Next-generation sequencing analysis of lager brewing yeast strains reveals the evolutionary history of interspecies hybridization. DNA Res. 2016:23:67–80. 10.1093/dnares/dsv037. 26732986 PMC4755528

[evaf004-B87] Paradis E, Claude J, Strimmer K. APE: analyses of phylogenetics and evolution in R language. Bioinformatics. 2004:20:289–290. 10.1093/bioinformatics/btg412. 14734327

[evaf004-B88] Peccoud J, Cordaux R, Gilbert C. Analyzing horizontal transfer of transposable elements on a large scale: challenges and prospects. Bioessays. 2018:40:1700177. 10.1002/bies.v40.2. 29283188

[evaf004-B89] Peccoud J, Loiseau V, Cordaux R, Gilbert C. Massive horizontal transfer of transposable elements in insects. Proc Natl Acad Sci U S A. 2017:114:4721–4726. 10.1073/pnas.1621178114. 28416702 PMC5422770

[evaf004-B90] Peris D, Langdon QK, Moriarty RV, Sylvester K, Bontrager M, Charron G, Leducq J-B, Landry CR, Libkind D, Hittinger CT. Complex ancestries of lager-brewing hybrids were shaped by standing variation in the wild yeast *Saccharomyces eubayanus*. PLoS Genet. 2016:12:e1006155. 10.1371/journal.pgen.1006155. 27385107 PMC4934787

[evaf004-B91] Peris D, Ubbelohde EJ, Kuang MC, Kominek J, Langdon QK, Adams M, Koshalek JA, Hulfachor AB, Opulente DA, Hall DJ, et al Macroevolutionary diversity of traits and genomes in the model yeast genus Saccharomyces. Nat Commun. 2023:14:690. 10.1038/s41467-023-36139-2. 36755033 PMC9908912

[evaf004-B92] Peter J, Chiara MD, Friedrich A, Yue J-X, Pflieger D, Bergstrom A, Sigwalt A, Barre B, Freel K, Llored A, et al Genome evolution across 1,011 *Saccharomyces cerevisiae* isolates. Nature. 2018:556:339–344. 10.1038/s41586-018-0030-5. 29643504 PMC6784862

[evaf004-B93] Quinlan AR, Hall IM. BEDTools: a flexible suite of utilities for comparing genomic features. Bioinformatics. 2010:26:841–842. 10.1093/bioinformatics/btq033. 20110278 PMC2832824

[evaf004-B94] Ramazzotti M, Stefanini I, Paola MD, Filippo CD, Rizzetto L, Berná L, Dapporto L, Rivero D, Tocci N, Weil T, et al Population genomics reveals evolution and variation of *Saccharomyces cerevisiae* in the human and insects gut. Environ Microbiol. 2019:21:50–71. 10.1111/emi.2019.21.issue-1. 30246283

[evaf004-B95] Revell LJ . phytools: an R package for phylogenetic comparative biology (and other things). Methods Ecol Evol. 2012:3:217–223. 10.1111/mee3.2012.3.issue-2.

[evaf004-B96] Roeder GS, Fink GR. Movement of yeast transposable elements by gene conversion. Proc Natl Acad Sci U S A. 1982:79:5621–5625. 10.1073/pnas.79.18.5621. 6291054 PMC346956

[evaf004-B97] Salazar AN, Gorter de Vries AR, van den Broek M, Brouwers N, de la Torre Cortes P, Kuijpers NGA, G. Daran J-M, Abeel T. Chromosome level assembly and comparative genome analysis confirm lager-brewing yeasts originated from a single hybridization. BMC Genomics. 2019:20:916. 10.1186/s12864-019-6263-3. 31791228 PMC6889557

[evaf004-B98] Salzberg LI, Martos AAR, Lombardi L, Jermiin LS, Blanco A, Byrne KP, Wolfe KH. A widespread inversion polymorphism conserved among Saccharomyces species is caused by recurrent homogenization of a sporulation gene family. PLoS Genet. 2022:18:e1010525. 10.1371/journal.pgen.1010525. 36441813 PMC9731477

[evaf004-B99] Sarilar V, Bleykasten-Grosshans C, Neuveglise C. Evolutionary dynamics of hAT DNA transposon families in Saccharomycetaceae. Genome Biol Evol. 2015:7:172–190. 10.1093/gbe/evu273. PMC4316626 25532815

[evaf004-B100] Scannell DR, Zill OA, Rokas A, Payen C, Dunham MJ, Eisen MB, Rine J, Johnston M, Hittinger CT. The awesome power of yeast evolutionary genetics: new genome sequences and strain resources for the Saccharomyces sensu stricto genus. G3 (Bethesda). 2011:1(1):11–25. 10.1534/g3.111.000273. 22384314 PMC3276118

[evaf004-B101] Schaack S, Gilbert C, Feschotte C. Promiscuous DNA: horizontal transfer of transposable elements and why it matters for eukaryotic evolution. Trends Ecol Evol. 2010:25(9):537–546. 10.1016/j.tree.2010.06.001. 20591532 PMC2940939

[evaf004-B102] Schliep KP . phangorn: phylogenetic analysis in R. Bioinformatics. 2011:27(4):592–593. 10.1093/bioinformatics/btq706. 21169378 PMC3035803

[evaf004-B103] Shen W, Le S, Li Y, Hu F. SeqKit: a cross-platform and ultrafast toolkit for FASTA/Q file manipulation. PLoS One. 2016:11:e0163962. 10.1371/journal.pone.0163962. 27706213 PMC5051824

[evaf004-B104] Skelly DA, Merrihew GE, Riffle M, Connelly CF, Kerr EO, Johansson M, Jaschob D, Graczyk B, Shulman NJ, Wakefield J, et al Integrative phenomics reveals insight into the structure of phenotypic diversity in budding yeast. Genome Res. 2013:23:1496–1504. 10.1101/gr.155762.113. 23720455 PMC3759725

[evaf004-B105] Song G, Dickins BJA, Demeter J, Engel S, Gallagher J, Choe K, Dunn B, Snyder M, Cherry JM. AGAPE (Automated Genome Analysis PipelinE) for pan-genome analysis of *Saccharomyces cerevisiae*. PLoS One. 2015:10:e0120671. 10.1371/journal.pone.0120671. 25781462 PMC4363492

[evaf004-B106] Stamatakis A . RAxML version 8: a tool for phylogenetic analysis and post-analysis of large phylogenies. Bioinformatics. 2014:30:1312–1313. 10.1093/bioinformatics/btu033. 24451623 PMC3998144

[evaf004-B107] Steinbiss S, Willhoeft U, Gremme G, Kurtz S. Fine-grained annotation and classification of de novo predicted LTR retrotransposons. Nucleic Acids Res. 2009:37:7002–7013. 10.1093/nar/gkp759. 19786494 PMC2790888

[evaf004-B108] Strope PK, Skelly DA, Kozmin SG, Mahadevan G, Stone EA, Magwene PM, Dietrich FS, McCusker JH. The 100-genomes strains, an *S. cerevisiae* resource that illuminates its natural phenotypic and genotypic variation and emergence as an opportunistic pathogen. Genome Res. 2015:25:762–774. 10.1101/gr.185538.114. 25840857 PMC4417123

[evaf004-B109] Stucka R, Lochmäller H, Feldmann H. Ty4, a novel low-copy number element in *Saccharomyces cerevisiae*: one copy is located in a cluster of Ty elements and tRNA genes. Nucleic Acids Res. 1989:17:4993–5002. 10.1093/nar/17.13.4993. 2548153 PMC318089

[evaf004-B110] Stucka R, Schwarzlose C, Lochmüller H, Häcker U, Feldmann H. Molecular analysis of the yeast Ty4 element: homology with Ty1, copia, and plant retrotransposons. Gene. 1992:122:119–128. 10.1016/0378-1119(92)90039-R. 1333437

[evaf004-B111] Wallau GL, Ortiz MF, Loreto ELS. Horizontal transposon transfer in eukarya: detection, bias, and perspectives. Genome Biol Evol. 2012:4:801–811. 10.1093/gbe/evs055. PMC3516303 22798449

[evaf004-B112] Wallau GL, Vieira C, Loreto ELS. Genetic exchange in eukaryotes through horizontal transfer: connected by the mobilome. Mob DNA. 2018:9(1):6. 10.1186/s13100-018-0112-9. 29422954 PMC5791352

[evaf004-B113] Wang Q-M, Liu W-Q, Liti G, Wang S-A, Bai F-Y. Surprisingly diverged populations of Saccharomyces cerevisiae in natural environments remote from human activity. Mol Ecol. 2012:21(22):5404–5417. 10.1111/mec.2012.21.issue-22. 22913817

[evaf004-B114] Wells JN, Feschotte C. A field guide to eukaryotic transposable elements. Annu Rev Genet. 2020:54(1):539–561. 10.1146/genet.2020.54.issue-1. 32955944 PMC8293684

[evaf004-B115] Xia W, Nielly-Thibault L, Charron G, Landry CR, Kasimer D, Anderson JB, Kohn LM. Population genomics reveals structure at the individual, host-tree scale and persistence of genotypic variants of the undomesticated yeast *Saccharomyces paradoxus* in a natural woodland. Mol Ecol. 2017:26(4):995–1007. 10.1111/mec.2017.26.issue-4. 27988980

[evaf004-B116] Yu G, Smith DK, Zhu H, Guan Y, Lam TT-Y. ggtree: an r package for visualization and annotation of phylogenetic trees with their covariates and other associated data. Methods Ecol Evol. 2017:8(1):28–36. 10.1111/mee3.2017.8.issue-1.

[evaf004-B117] Yue J-X, Li J, Aigrain L, Hallin J, Persson K, Oliver K, Bergstrom A, Coupland P, Warringer J, Lagomarsino MC, et al Contrasting evolutionary genome dynamics between domesticated and wild yeasts. Nat Genet. 2017:49(6):913–924. 10.1038/ng.3847. 28416820 PMC5446901

[evaf004-B118] Zheng X, Levine D, Shen J, Gogarten SM, Laurie C, Weir BS. A high-performance computing toolset for relatedness and principal component analysis of SNP data. Bioinformatics. 2012:28(24):3326–3328. 10.1093/bioinformatics/bts606. 23060615 PMC3519454

[evaf004-B119] Zhu YO, Sherlock G, Petrov DA. Whole genome analysis of 132 clinical *Saccharomyces cerevisiae* strains reveals extensive ploidy variation. G3 (Bethesda). 2016:6(8):2421–2434. 10.1534/g3.116.029397. 27317778 PMC4978896

